# Genome-wide analysis of cytochrome P450 genes in *Citrus clementina* and characterization of a CYP gene encoding flavonoid 3′-hydroxylase

**DOI:** 10.1093/hr/uhac283

**Published:** 2022-12-23

**Authors:** Xiaojuan Liu, Qin Gong, Chenning Zhao, Dengliang Wang, Xianming Ye, Guixia Zheng, Yue Wang, Jinping Cao, Chongde Sun

**Affiliations:** Laboratory of Fruit Quality Biology, The State Agriculture Ministry Laboratory of Horticultural Plant Growth, Development and Quality Improvement, Zhejiang Provincial Key Laboratory of Integrative Biology of Horticultural Plants, Zhejiang University, Hangzhou, China; Laboratory of Fruit Quality Biology, The State Agriculture Ministry Laboratory of Horticultural Plant Growth, Development and Quality Improvement, Zhejiang Provincial Key Laboratory of Integrative Biology of Horticultural Plants, Zhejiang University, Hangzhou, China; Laboratory of Fruit Quality Biology, The State Agriculture Ministry Laboratory of Horticultural Plant Growth, Development and Quality Improvement, Zhejiang Provincial Key Laboratory of Integrative Biology of Horticultural Plants, Zhejiang University, Hangzhou, China; Institute of Fruit Tree Research, Quzhou Academy of Agriculture and Forestry Acience, Quzhou, China; Research and Development Department, Zhejiang Jianong Fruit &Vegetable Co., Ltd, Quzhou, China; Research and Development Department, Zhejiang Jianong Fruit &Vegetable Co., Ltd, Quzhou, China; Laboratory of Fruit Quality Biology, The State Agriculture Ministry Laboratory of Horticultural Plant Growth, Development and Quality Improvement, Zhejiang Provincial Key Laboratory of Integrative Biology of Horticultural Plants, Zhejiang University, Hangzhou, China; Laboratory of Fruit Quality Biology, The State Agriculture Ministry Laboratory of Horticultural Plant Growth, Development and Quality Improvement, Zhejiang Provincial Key Laboratory of Integrative Biology of Horticultural Plants, Zhejiang University, Hangzhou, China; Laboratory of Fruit Quality Biology, The State Agriculture Ministry Laboratory of Horticultural Plant Growth, Development and Quality Improvement, Zhejiang Provincial Key Laboratory of Integrative Biology of Horticultural Plants, Zhejiang University, Hangzhou, China

## Abstract

Cytochrome P450s (CYPs) are the largest family of enzymes in plant and play multifarious roles in development and defense but the available information about the CYP superfamily in citrus is very limited. Here we provide a comprehensive genome-wide analysis of the CYP superfamily in *Citrus clementina* genome, identifying 301 CYP genes grouped into ten clans and 49 families. The characteristics of both gene structures and motif compositions strongly supported the reliability of the phylogenetic relationship. Duplication analysis indicated that tandem duplication was the major driving force of expansion for this superfamily. Promoter analysis revealed numerous *cis*-acting elements related to various responsiveness. RNA-seq data elucidated their expression patterns in citrus fruit peel both during development and in response to UV-B. Furthermore, we characterize a UV-B-induced CYP gene (*Ciclev10019637m*, designated *CitF3′H*) as a flavonoid 3′-hydroxylase for the first time. CitF3′H catalyzed numerous flavonoids and favored naringenin in yeast assays. Virus-induced silencing of *CitF3′H* in citrus seedlings significantly reduced the levels of 3′-hydroxylated flavonoids and their derivatives. These results together with the endoplasmic reticulum-localization of CitF3′H in plant suggest that this enzyme is responsible for the biosynthesis of 3′-hydroxylated flavonoids in citrus. Taken together, our findings provide extensive information about the CYP superfamily in citrus and contribute to further functional verification.

## Introduction

Cytochromes P450s (CYPs) superfamily constitute the largest family of enzymes in plants, accounting for about 1% of the protein-coding genes in several flowering plants [1]. All plant CYPs share a common heme-thiolate catalytic center and are membrane-bound, usually anchored on the endoplasmic reticulum (ER). The CYP superfamily catalyzes a mono-oxygenation reaction of a carbon atom to form ketones, alcohols, etc., which contributes to further chemical expansion through *O*-methylation, *O*-acylation, *O*-glycosylation, etc. [2]. Plant CYPs were initially classified into two clades: the A-type (plant-specific) and the non-A-type (non-plant-specific) [3]. Subsequently, the concept of CLANS (higher order groupings of CYP families) was introduced into the classification system and is extensively adopted nowadays. The initial defined A-type became clan 71 and the non-A-type consisted of the other clans [4], for example, there were ten clans (clans 51, 72, 74, 85, 86, 97, 710, 711, 727, and 746) belonging to the non-A-type in land plants [1]. CYPs are named by the CYP nomenclature system based on homology and phylogenetic relationship [5], a typical CYP name includes a number indicating the CYP family and a letter after the number designating the subfamily [6].

Over the past decades, the number of plant CYPs has increased rapidly, and more than 32 000 plant CYPs have been named so far, of which over 800 CYPs have been functionally characterized [2]. Plant CYPs are involved in numerous biochemical pathways and play diverse roles in development and defense (e.g. UV irradiation, dehydration, and pathogens) [2, 7, 8]. The biochemical reactions include the biosynthesis of sterols (e.g. CYP51 and CYP710), carotenoids (e.g. CYP97), amino acids (e.g. CYP79), fatty acids (e.g. CYP86), phenylpropanoids (e.g. CYP73, CYP98 and CYP84), flavonoids (e.g. CYP75 and CYP93), coumarins (e.g. CYP71 and CYP82), terpenoids (e.g. CYP76 and CYP706), alkaloids (e.g. CYP80 and CYP719), plant hormones (CYP74 and CYP90), etc. [2, 9–11]. Plant CYPs usually have high substrate promiscuity and classifying these genes into the correct family or subfamily would be of great help for functional prediction [2]. The advancements in genome sequencing have underlain the identification, classification and functional elucidation of the CYP superfamily in different species. To date, the genome-wide analysis of CYP genes has been performed in some plant species, such as *Arabidopsis thaliana* [12], *Lonicera japonica* [13], *Oryza sativa* [14], *Vitis vinifera* [15], *Sorghum bicolor* [16], and *Glycine max* [17].

Citrus is one of the most important fruit crops worldwide, and citrus fruit, especially the flavedo is rich in a vast array of primary and secondary metabolites, such as terpenes and flavonoids [18]. The largest enzymatic superfamily, CYP genes, are considered as key enzymes in the biosynthesis of these metabolites and play critical roles in the development and defense of citrus. However, only several CYP genes have been functionally characterized in citrus, including one gene from *Citrus unshiu* (*CYP97C27*) encoding a carotenoid epsilon-ring hydroxylase [19], two genes (*CYP71CD1* and *CYP71BQ4*) from *Citrus sinensis* involved in protolimonoid biosynthesis [20], one gene (*CYP82D64*) from *Citrus paradisi* and its orthologous gene from *Citrus hystrix* function as xanthotoxin 5-hydroxylases [21], one gene (*CYP93A65*) from *Fortunella crassifolia* encoding a flavone synthase [22]. Previously, the exon-intron organization, classification and phylogenetic relationship of CYP genes were reported in three citrus species [23]. However, these results were confusing because the CYP genes were not assigned proper CYP names and classifications by the CYP nomenclature system when they were published [23]. Although the names and classification of these genes were corrected later in a statement on the Cytochrome P450 Homepage [5], a more comprehensive and rigorous analysis of the CYP genes in citrus is still indispensable for further research.

In this study, we performed a comprehensive analysis of the CYP superfamily based on the latest version of *Citrus clementina* genome [24] and identified 301 genes encoding 319 proteins. The present study includes their phylogenetic relationships, conserved motifs, gene structures, gene duplications, promoter *cis*-acting elements as well as their expression profiles in citrus fruit peel during development and in response to UV-B irradiation. Furthermore, we report the identification and characterization of a UV-B-induced CYP, a flavonoid 3′-hydroxylase (designated CitF3′H) in citrus. Substrate specificity in yeast assays, virus-induced gene silencing (VIGS) and subcellular localization assays together confirmed its role in enhancing the accumulation of 3′-hydroxylated flavonoids in citrus. The current study will provide a wealth of valuable information for a better understanding of the CYP genes in citrus and lay the foundation for the functional characterization of plant CYP genes.

## Results

### The *Citrus clementina* genome contains 301 CYP genes

**Figure 1:**
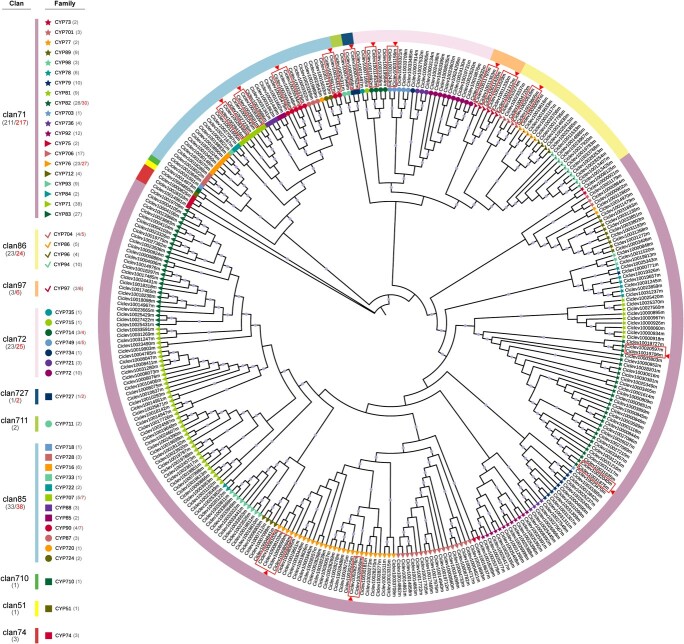
Phylogenetic analysis of CYP genes in *Citrus clementina*. The protein sequences of citrus CYPs were used to construct a maximum likelihood tree with 5000 bootstrap replicates. Bootstrap values greater than 0.7 are indicated by a gray circle in the middle of each branch. Clans and families are indicated using color strips and symbols, respectively. Alternative splicing transcripts from the same gene are indicated with red boxes, where the longest transcript is indicated with an arrow.

Based on the latest version of *C. clementina* genome (v1.0), a total of 319 protein sequences were identified as CYP superfamily members in combination with hmmsearch, local BLSATP search and domain verification approaches (Fig. 1; Fig. S1 and Table S1, see online supplementary material). These CYP proteins were eventually found to be encoded by 301 genes due to the alternative splicing events. Analysis of physical and chemical properties showed that the characteristics of the 319 citrus CYP proteins varied widely, with protein lengths (number of amino acids) ranging from 303 to 621, molecular weights ranging from 34.07 to 71.09 kDa and theoretical isoelectric points ranging from 4.91 to 9.69. The prediction of subcellular localization showed that most of the citrus CYP proteins (292 of 319) were localized in the endomembrane system, followed by the organelle membrane (22 of 319). Other properties of CYPs, including the instability index, aliphatic index, and grand average of hydropathicity are also provided in Table S1 (see online supplementary material).

### Phylogenetic relationship of CYPs in citrus

To analyse the phylogenetic relationships of CYPs from *C. clementina*, an unrooted ML tree was inferred from a trimmed alignment of 319 citrus CYP protein sequences. These citrus CYPs were further assigned to specific families and clans based on the systematic names designated by the CYP nomenclature system [5] (Fig. 1; Table S1 and S2, see online supplementary material). As results, the family and clan of citrus CYPs matched well with the phylogenetic clades, which in turn indicated the reliability of the phylogenetic tree.

A total of ten CYP clans (49 families) were recognized in citrus, and clans could be further divided into two distinct clades (A-type and non-A-type) based on the phylogenetic tree (Fig. 1) [3]. Clan 71 belonged to the A-type, while the other nine clans all belonged to the non-A-type. Among the non-A-type, clans 72, 86, and 97 were grouped into one cluster; clans 51, 85, 710, 711, and 727 formed another cluster; clan 74 constituted a single-clan cluster. Clans 71, 72, 85, and 86 were multi-family clans and included 20 families (211 genes), seven families (23 genes), 12 families (33 genes), and four families (23 genes), respectively. The remaining six clans (clans 51, 74, 97, 710, 711, and 727) were single-family clans and up to three genes were included in each clan.

### Conserved motifs and gene structures of CYPs in citrus

**Figure 2:**
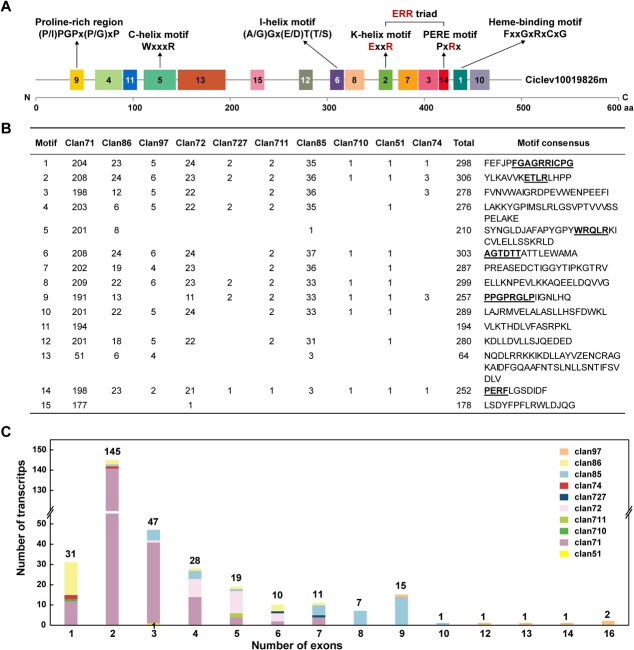
Distribution of conserved motifs and exons in ten clans of citrus CYPs. **A** Schematic diagram of 15 conserved motifs in citrus CYP proteins, taking Ciclev10019826m as an example, the signature motifs containing functionally characterized domains are indicated. N and C represent the N-terminal and C-terminal, respectively. **B** Distribution of 15 conserved motifs in ten CYP clans. **C** Exon distribution of CYP transcripts in ten CYP clans.

A total of 15 conserved motifs were identified in citrus CYP proteins using MEME software (Fig. 2A and B; Fig. S2 and S3, see online supplementary material). In general, the composition of these motifs showed a considerable divergence between the A-type and non-A-type CYPs while similar patterns were found within the same CYP clan. All of the 15 motifs were recognized in the A-type CYPs, while few motifs (5 to13) were recognized in the non-A-type CYPs (Fig. 2A). The majority of motifs were conserved in citrus CYPs, including nine motifs located on the C-terminal (motifs 1, 2, 3, 6, 7, 8, 10, 12, and 14) and two motifs (4 and 9) located on the N-terminal. Of the 11 conserved motifs, five of them (motifs 1, 2, 6, 9, and 14) contained the functionally characterized domains (Fig. 2B) [25, 26]. Motif 1 contained the core catalytic center, heme-binding motif (FxxGxRx**C**xG), in which the cysteine (C) was the axial ligand to the thiolate heme; motifs 2 and 14 contained the K-helix motif (**E**xx**R**) and PERF motif (Px**R**x), respectively. The E and R residues of the K-helix and the R residue of PERF motif formed the E-R-R triad, which was thought to stabilize the highly conserved three-dimensional structure; motif 6 contained the consensus (A/G)Gx(E/D)T(T/S) of the I-helix motif, which was involved in oxygen binding and activation; motif 9 contained a proline-rich region with the consensus of (P/I)PGPx(P/G)xP, this region was considered as a membrane hinge that was crucial for correct orientation of CYP enzyme to the membrane. Nevertheless, the remaining four motifs (motifs 5, 11, 13, and 15), which were located on the N-terminal, were not conserved in all citrus CYPs. The absence of these motifs was common in the non-A-type CYPs. One of these motifs has been functionally elucidated, i.e. motif 5 contained the C-helix motif (WxxxR), and the W and R residues contributed to the interaction with a propionate side chain of the heme [27].

The organizations of exon, intron, coding sequences (CDS) and untranslated region (UTR) were also summarized to better understand the structure of citrus CYP transcripts (Fig. 2C; Fig. S4, see online supplementary material). The CDS-UTR composition was highly variable in the non-A-type CYPs compared with that of the A-type CYPs, whereas the gene structure within the identical CYP family was semblable (Fig. S4, see online supplementary material). The A-type CYPs possessed one to seven exons, of which 65% (141 out of 217) possessed two exons. The non-A-type CYPs possessed a more variable number of exons, ranging from one to 16, for example, clan 51 possessed three exons, clan 72 possessed three to six exons and clan 97 possessed nine to 16 exons. Overall, the exon number of citrus CYPs varied widely, ranging from one to 16. A total of 31 CYPs with single exons, 145 CYPs (approximately half of the CYPs) with two exons, 47 CYPs with three exons, 28 CYPs with four exons, 62 CYPs with five to nine exons and six CYPs with ten to 16 exons were found in citrus CYPs (Fig. 2C).

Taken together, the patterns of conserved motifs and gene structures varied considerably between the A-type and non-A-type CYPs; however, similar patterns were observed within the same clan or family which enhanced the credibility of the phylogenetic relationship and group classification.

### *Citrus* CYP genes exhibit prevalent gene duplication events

**Figure 3:**
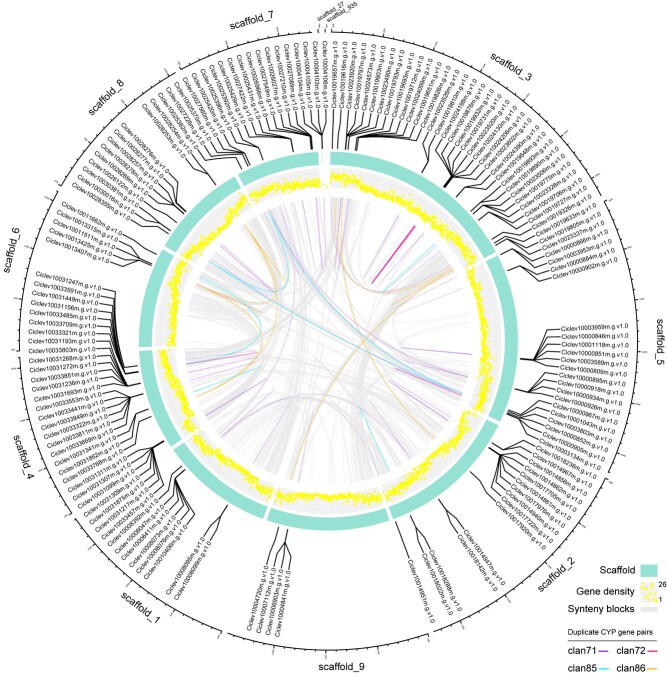
Clan-specific gene duplication among citrus CYP genes. Duplicated gene pairs (Table S4) were visualized in the circle and linked with clan-specific colors as in Fig. 1. Segmental duplicated gene pairs are positioned on different citrus scaffolds while tandem duplicated gene pairs are positioned within the same scaffolds (present as incomplete links). Light gray lines in the circle indicate all syntenic blocks in the *Citrus clementina* genome. The outer track (light cyan) indicates different scaffolds, and the inner track (yellow scatter diagram) indicates gene density (number of genes per 0.1 Mb).

To investigate the gene duplication events in citrus CYPs, a collinearity and gene duplication analysis within *C. clementina* genome were carried out using the MCScanX algorithm. Firstly, the origins of duplicated CYP genes were classified into four duplication events (tandem, proximal, WGD/segmental and dispersed). The majority of CYP genes (41.2%, 124 out of 301) were duplicated from the tandem event, compared with 32.2% (97) from dispersed, 15.9% (48) from proximal and 10.6% (32) from WGD/segmental (Table S3, see online supplementary material). These results showed that tandem duplication seemed to be the major driving force for expanded families of citrus CYPs. Moreover, pairwise collinear blocks were generated to identify the segmentally and tandemly duplicated gene pairs in the citrus CYPs (Fig. 3; Table S4, see online supplementary material). The results demonstrated that the duplicated gene pairs occurred within four CYP clans (clans 71, 72, 85, and 86), including 20 gene pairs of segmental duplication and 82 gene pairs of tandem duplication. Among them, 13 CYP genes within two clans (clans 71 and 86) exhibited both segmental and tandem duplication events. These results together suggested that both duplication events might be responsible for the expansion of CYP genes in citrus. Furthermore, the citrus CYP gene superfamily was likely to have undergone purifying selection, because almost all duplicated CYP gene pairs had Ka/Ks ratios less than one (Table S4, see online supplementary material). Additionally, the physical locations of all CYP genes were mapped to scaffolds of *C. clementina* genome, these genes were unevenly distributed across 11 scaffolds, with most (296 out of 301) genes located on nine scaffolds (scaffold_1 to scaffold_9) (Fig. S5, see online supplementary material).

### Analysis of *cis*-acting elements in the promoter region of citrus CYPs

**Figure 4:**
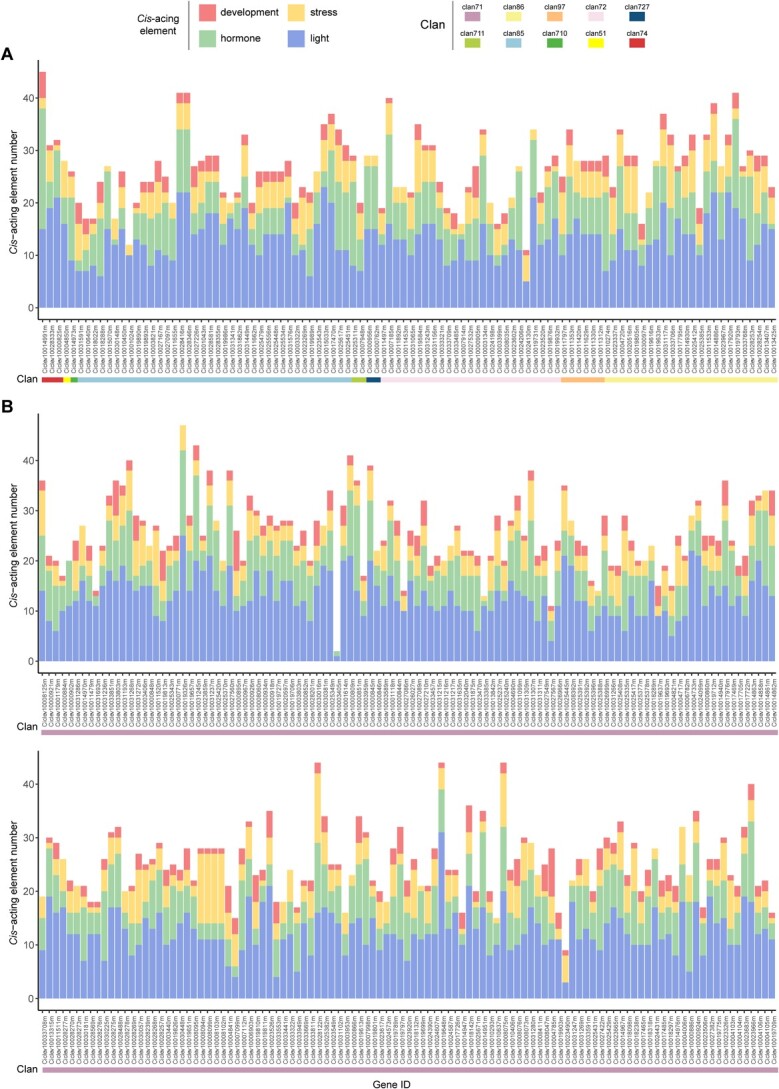
Number of *cis*-acting elements in the promoter region of ten clans of citrus CYPs. The predicted *cis*-acting elements of citrus CYPs belonging to clan 71 **(A)** and the other nine clans **(B) **were classified into four broad categories, including development, stress, hormone, and light responsiveness.

The 2000-bp region upstream of the initiation codon (ATG) of each citrus CYP transcript was regarded as the promoter sequence in this study and subjected to the PlantCARE database for the prediction of *cis*-regulatory elements. The predicted *cis*-acting elements other than the core elements could be classified into four broad categories based on their responsiveness to any perturbation, including development, stress, hormone and light responsiveness (Fig. 4; Figs S6 and S7,Table S5, see online supplementary material). There was no obvious difference among different clans; almost all of the citrus CYPs possessed the four types of *cis*-acting elements analysed above. Light-responsive elements were found to be the most prevalent in the promoter regions of citrus CYPs (Fig. 4). A sum of 35 light-responsive elements was predicted, and the five elements with the highest frequency were as follows: Box 4 (95.6%, 305 out of 319), G-box (85.2%), GT1-motif (63.6%), TCT-motif (57.7%), and GATA-motif (51.7%). Hormone-responsive elements were also detected, including the most frequently occurred (265 out of 319) abscisic acid (ABA)-responsive element (ABRE), the auxin-responsive elements (e.g. TGA-element and AuxRR-core), the methyl jasmonate (MeJA)-responsive element (TGACG-motif), the gibberellin-responsive elements (e.g. P-box and TATC-box) and the salicylic acid-responsive elements (e.g. TCA-element). Several stress-responsive elements (e.g. ARE, MBS, and LTR) that were related to anaerobic, drought and low-temperature responsiveness were found in 250 (78.4%), 171 (53.6%), and 140 (43.9%) citrus CYPs, respectively. In addition, some development-responsive elements (e.g. the CAT-box, O2-site, GCN4_motif, and circadian) that were associated with meristem expression, zein metabolism regulation, endosperm expression, and circadian control, were identified in 135 (42.3%), 113 (35.4%), 69 (21.6%), and 59 (18.5%) citrus CYPs. These results showed that these abundant *cis*-acting elements might regulate the expression of citrus CYPs during development and in response to light, stress, and hormones.

### Expression profiling of CYP genes in citrus fruit peel during development and in response to UV-B treatment

The transcriptome data in the flavedo of citrus during developmental stages were analysed [28]. Out of the 319 CYP transcripts, 271 (85.0%) were expressed in at least one developmental stage, with the FPKM values ranging from 0.01 to 702.2 (Table S1, see online supplementary material). The expressed CYP genes were further clustered based on their expression patterns using Mfuzz and grouped into nine distinct expression clusters. Each cluster contains a set of genes with similar expression patterns ranging from 22 to 46 members (Fig. S8,Table S1, see online supplementary material).

Light (e.g. UV light) plays a critical role in plant growth and defense induction [29]. As shown in Fig. 4, a large number of light-responsive *cis*-acting elements were observed in citrus CYPs, and several *cis*-acting elements (e.g. G-box and MRE) have been reported to be involved in UV-B responsiveness [30, 31]. To understand how citrus CYPs responded to UV-B treatment, the RNA-Seq data of the citrus flavedo which was directly irradiated by UV-B were analysed [28]. The CYPs could be roughly divided into four groups based on their response to UV-B treatment, and the A group consisted of 74 CYP transcripts, which were up-regulated in the flavedo after UV-B irradiation compared to the control group (Fig. S9, see online supplementary material). These up-regulated members might play important roles in the protective response to UV-B irradiation in citrus.

### Identification of a CYP gene encoding putative flavonoid 3′-hydroxylase in citrus

**Figure 5:**
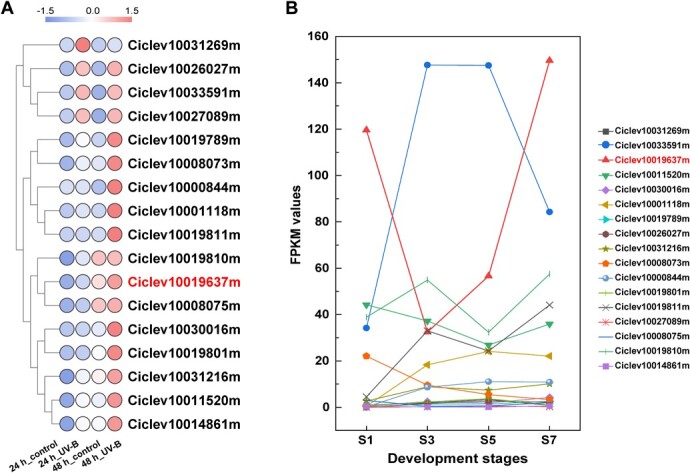
Expression pattern of 17 UV-B-induced CYP genes belonging to flavonoid-related families in the flavedo of citrus both in response to UV-B irradiation (**A**) and during development (**B**). The mean expression values of each gene in response to UV-B irradiation were automatically scaled and visualized as heatmaps by TBtools [45]. Expression values were obtained from our previous study [28] and can be found in Table S1.

It has been well documented that plant CYPs include key enzymes in the biosynthesis of UV-B protectants, such as flavonoids [9]. For example, several members belonging to CYP families 71, 75, 82, 93, 98, and 706 have been proven to be vital for flavonoid biosynthesis [22, 32–35]. In this study, 17 CYP genes belonging to flavonoid-related families were found to be up-regulated in the flavedo of citrus after irradiation by UV-B (Fig. 5A; Fig. S9, see online supplementary material). Among the 17 up-regulated genes, one CYP gene (*Ciclev100019637m*), designated *CYP75B81* by the CYP nomenclature system, showed the highest expression level at the early stage (S1) when the citrus flavonoids were rapidly biosynthesized [28] (Fig. 5B). Moreover, this gene was predicted to encode a citrus flavonoid 3′-hydroxylase (termed CitF3′H) because it belongs to the CYP75B subfamily, most members of which hydroxylate the 3′ position of flavonoids in plant [33]. Therefore, this CYP gene was speculated to be involved in flavonoid biosynthesis both during development and in response to UV-B irradiation. Another up-regulated gene *Ciclev10033591m* (*CYP71AS15*) also exhibited relatively high transcript levels in the flavedo of citrus during development (Fig. 5B). However, the function of the CYP71AS subfamily was still unclear, and yeast assays showed that there was no detectable product when two representative flavonoids (naringenin and apigenin) acted as substrates (Fig. S10, see online supplementary material). Hence, the catalytic activity of this enzyme needs to be clarified further.

### Substrate specificity of *CitF3′H* in a yeast system

**Figure 6:**
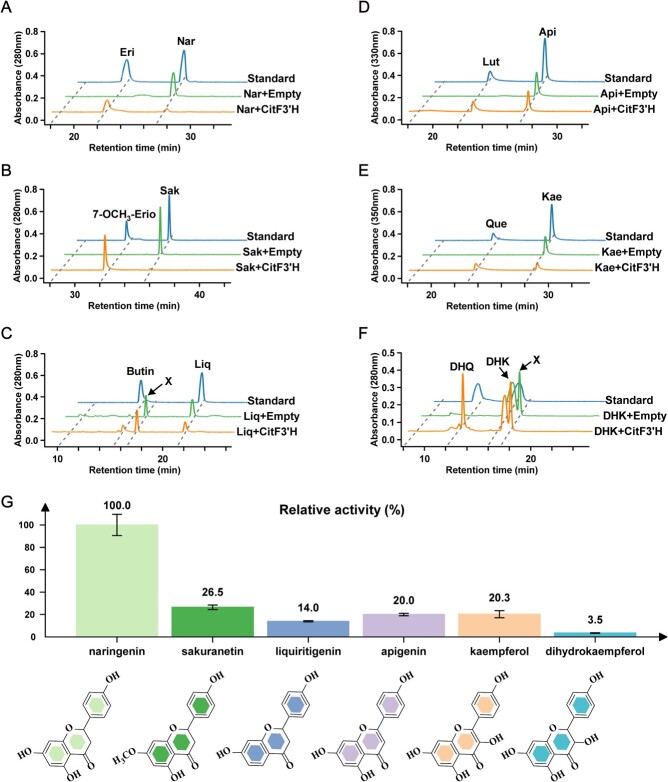
Characterization of CitF3′H enzyme activity in a yeast system. (**A**–**F**) HPLC chromatograms of yeast cultures incubated with naringenin (**A**), sakuranetin (**B**), liquiritigenin (**C**), apigenin (**D**), kaempferol (**E**), and dihydrokaempferol (**F**). Top, authentic compounds of substrates and their 3′-hydroxylated products; middle, yeast harboring the empty vector; bottom, yeast expressing CitF3′H. Nar, naringenin; Eri, eriodictyol; Sak, sakuranetin; 7-OCH3-Erio, 7-*O*- methyleriodictyol; Liq, liquiritigenin; Butin, Butin; Api, apigenin; Lut, luteolin; Kae, kaempferol; Que, quercetin; DHK, dihydrokaempferol; DHQ, dihydroquercetin; X, unknown peak. MS/MS data of the new peaks produced by CitF3′H and their authentic compounds are indicated in Fig. S11. **G** Relative activity of CitF3′H with different substrates. Values are means ±SE (*n* = 3). Structural formulas of substrates are indicated below.

To investigate the substrate specificity of CitF3′H, the putative flavonoids substrates (flavanones, flavones, flavonols, and dihydroflavonols) were added to the medium of yeast strains harboring the *CitF3′H-*pYES2/NT C, with the empty vector as a control. The reaction mixture was analysed using HPLC and MS/MS, and the generated products were identified by comparing them with the corresponding authentic standards (Fig. 6; Fig. S11, see online supplementary material).

The flavanones naringenin, sakuranetin, liquiritigenin, pinocembrin, isosakuranetin, and naringenin glycosides (naringin and narirutin) were tested. Naringenin, sakuranetin, and liquiritigenin were all converted to their expected 3′-hydroxylated product by the yeast strains expressing *CitF3′H* compared with the empty vector (Fig. 6A–C). However, two naringenin glycosides naringin and narirutin (naringenin 7-*O*-neohesperidoside and naringenin 7-*O*-rutinoside) could no longer be catalyzed by this enzyme. Pinocembrin and isosakuranetin, which lack a free hydroxyl group at the 4′ position on the B-ring, could not be catalyzed either (Fig. S12, see online supplementary material).

Several flavones, including apigenin, chrysin, genkwanin, baicalein, scutellarein, norwogonin, and wogoninn, were tested. Out of the tested flavones, only apigenin could be catalyzed by CitF3′H, yielding its 3′-hydroxylated product (Fig. 6D). The flavones without any substituents on the B-ring (e.g. chrysin, baicalein, norwogonin, and wogonin) or with extra modifications on the basis of the 5,7-dihydroxyl groups on the A-ring (e.g. genkwanin and scutellarein) were not catalyzed by this enzyme (Fig. S12, see online supplementary material). With the flavonol (kaempferol) and dihydroflavonol (dihydrokaempferol) as substrates, their 3′-hydroxylation products (quercetin and dihydroquercetin) were produced by CitF3′H (Fig. 6E and F).

Relative activities toward different flavonoid substrates were determined in order to investigate the substrate preference of CitF3′H. The results showed that CitF3′H exhibited the highest relative activity (100%) toward naringenin compared with less than 30% toward other substrates (sakuranetin, liquiritigenin, apigenin, kaempferol, and dihydrokaempferol) (Fig. 6G). Taken together, CitF3′H was a flavonoid 3′-hydroxylase that preferred flavanone naringenin to other flavonoids in yeast assays.

### Silencing of *CitF3′H* leads to reduced 3′-hydroxylated flavonoids in citrus

**Figure 7:**
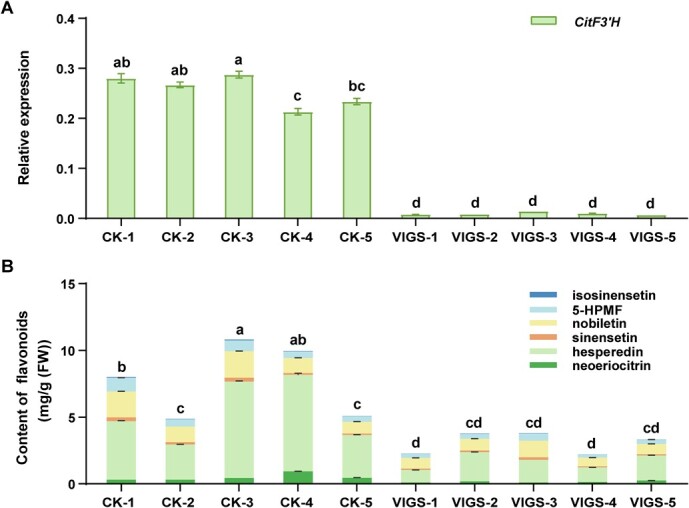
Virus-induced gene silencing (VIGS) of *CitF3′H* in citrus seedlings. **A** Relative expression of *CitF3’H* in gene-silenced citrus seedlings. **B** Contents of 3′-hydroxylated flavonoids and their derivatives in gene-silenced citrus seedlings. Stacked bars marked by different lowercase letters are significantly different (*P* < 0.05) following one-way ANOVA and Ducan’s test. CK, empty vector control plants; VIGS, *CitF3’H* gene silencing plants. Values are means ±SE (*n* = 3).

A VIGS system was used to silence *CitF3′H* in citrus seedlings in order to explore its function in flavonoid hydroxylation in planta. The transcript level of *CitF3′H* in five positive VIGS lines was significantly reduced by ~95% compared with that of control plants (infiltrated with empty vector) (Fig. 7A). Subsequently, the potential catalytic products of CitF3′H in citrus plants were measured, including two 3′-hydroxylated flavonoids, i.e. neoeriocitrin and hesperidin and four 3′-methoxylated flavonoids, i.e. sinensetin, isosinensetin, nobiletin, and 5-hydroxy-6,7,8,3′,4′-pentamethoxylflavone (5-HPMF). The results showed the total content of these flavonoids was substantially reduced by ~60% in the VIGS lines compared with that of control plants (Fig. 7B).

### Subcellular localization of *CitF3’H*

**Figure 8:**
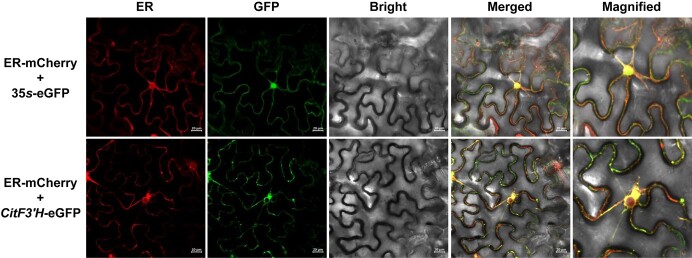
Subcellular localization of *CitF3’H* in tobacco leaves. Tobacco leaves were co-transformed with *CitF3’H*-green fluorescent (GFP) and mCherry-labeled endoplasmic reticulum (ER) marker protein by agroinfiltration. The corresponding GFP (empty vector) was used as a positive control. The constructs co-expressed in tobacco leaves are shown on the left side of each row, i.e. ER-mCherry+*35S*-eGFP and ER-mCherry+*CitF3’H*-GFP. Yellow pixels in the merged image indicate overlapping green and red fluorescence signals. Bars = 20 μm.

The *CitF3’H-*GFP construct and an ER-maker construct with mCherry-label were co-expressed in tobacco leaves to visualize the subcellular localization of *CitF3’H*. The non-targeted empty vector (GFP) displayed a diffuse localization throughout the tobacco cell, whereas the green signal of *CitF3’H-*GFP merged well with the red signal of mCherry-labeled ER marker in cells co-transformed with *CitF3’H-*GFP and the ER-marker (Fig. 8). These results indicated that *CitF3’H* was localized in ER, which was consistent with the predicted subcellular localization of endomembrane system (Table S1, see online supplementary material) and in line with the member-localization of most plant CYPs [26].

## Discussion

In this study, we carried out a comprehensive investigation of the CYP gene family members in *C. clementina* genome, including their phylogenetic relationships, conserved motifs, gene structures, gene duplication events, *cis*-acting elements, and gene expression patterns during citrus development and in response to UV-B irradiation. Additionally, we identified a citrus CYP gene (*Ciclev10019637m,* designated *CitF3’H*) responsive to UV-B irradiation as a flavonoid 3′-hydroxylase with a preference for flavanone naringenin in a yeast system. Subcellular localization assays in tobacco demonstrated the ER-localization of *CitF3’H* in plant and silencing of *CitF3’H* in citrus plant attenuated the hydroxylation of flavonoids at the 3′ position.

A total of 301 CYP genes grouped into 49 families were identified from *C. clementina* genome (Fig. 1; Table S1, see online supplementary material), of which five CYP genes were newly identified compared with the previous research [23]; this discrepancy was probably caused by genomic database updates. The CYP complement of *C. clementina* was typical in angiosperms which consisted of about 300 genes and 50 families. For example, 245 CYP (47 families) were identified from *A. thaliana* [12], 326 CYP genes (45 families) were identified from *O. sativa* [14], 236 CYP genes (47 families) were identified from *V. vinifera* [15] and 317 CYP genes (48 families) were identified from *G. max* [17].

The citrus CYP families were further grouped into ten clans, and four multi-family clans (clans 71, 72, 85, and 86) remained the four largest clans, which contributed 96% to the total number of CYP genes in citrus (Fig. 1). Similar to most Angiosperms, which underwent a burst gene duplication in order to match various adaptive requirements, intensive gene duplications within the four CYP clans were also observed in citrus (Fig. 3; Table S4, see online supplementary material) [2, 26, 36]. A sum of 20 CYP gene pairs of segmental duplication was observed in citrus. These genes were inferred to be caused by the ancient triplication WGD (γ event) during evolution as there were no recent WGDs with the exception of the γ event in citrus [37]. In addition to the segmental duplication events, more tandem duplication events (82 gene pairs) were observed, which suggested that the expansion of CYP genes in citrus was mainly through the mechanism of tandem duplication. These findings were consistent with the phenomenon found in grapevine where most CYP genes arose through tandem duplications [15].

Plant CYPs were divided into two types: the A-type (clan 71) and the non-A-type (the other clans) [4]. The non-A-type CYPs were more ancient than the A-type CYPs and were considered to have more time to undergo rearrangement and gene duplication, resulting in a more divergent structural organization than the A-type CYPs [38]. Likewise, among the CYP genes in citrus, the non-A-type CYPs tended to be more divergent than that of the A-type in terms of gene structures and conserved motifs (Fig. 2). Despite the great variation between the two types of CYPs, five motifs appeared to be conserved in both CYP types in citrus: the proline-rich region (motif 9), the I-helix motif (motif 6), the K-helix motif (motif 2), the PERE motif (motif 14), and the heme-binding motif (motif 1). These motifs have been suggested to be vital for the catalytic function of the CYP enzyme [25–27]. However, four motifs (5, 11, 13, and 15) on the N-terminal were absent in most of the non-A-type CYPs (Fig. 2A and B). The four motifs were common in the A-type CYPs and were assumed to play an important role in the A-type CYPs. Taken together, these structural diversifications in citrus CYPs may lead to a wide range of substrate specificities, resulting in varying physiological activities.

Much evidence has suggested that plant CYPs participate in kinds of biochemical pathways and play important roles in multiple biological progress, including development and stress response [2, 7, 8]. The presence of various *cis*-acting elements in the promoter region of citrus CYP genes also suggested that citrus CYPs were capable of responding to many perturbations in plant, especially the responsiveness to light irradiation because light-responsive elements appeared to be the most prevalent among the citrus CYPs (Fig. 4). The expression profiles of citrus CYPs were analysed in the flavedo of citrus both during development and in response to UV-B irradiation, and the results showed that the citrus CYPs could be clustered into different groups based on their expression patterns, and the genes within the same cluster might be involved in some related functions (Figs S8 and S9, see online supplementary material). For example, a set of CYP genes were up-regulated in the citrus flavedo exposure to UV-B irradiation, of which 17 CYP genes belonged to the flavonoid-related CYP families (CYP71, 75, 82, 93, 98, and 706) in plant [22, 32–35]. Therefore, the 17 CYP genes were speculated to be involved in the enhanced accumulation of UV-absorbing flavonoids. Of the 17 CYP genes, one gene (*Ciclev100019637m* termed as *CYP75B81*) showed the highest expression level at the early stage (S1) of citrus fruit development when flavonoids were rapidly biosynthesized (Fig. 5). Therefore, this gene was probably involved in the flavonoid biosynthesis both during development and in response to UV-B irradiation. Furthermore, this gene was predicted to be a putative flavonoid 3′-hydroxylase in citrus (CitF3’H) because most members in the CYP75B subfamily catalyzed the hydroxylation of flavonoids at the 3′-position [33].

Hesperidin, neoeriocitrin, sinensetin, isosinensetin, nobiletin, and 5-HPMF, the representative flavonoids in citrus, are all derived from 3′-hydroxylated flavonoids (e.g. eriodictyol and luteolin) [39]. Silencing of *CitF3’H* in the seedlings of citrus resulted in a significant reduction in the content of these flavonoids, of which hesperidin (hesperetin glycoside), the most abundant flavonoid in citrus, decreased the most by ~69% (Fig. 7B). Heterologous expression in yeast confirmed that CitF3’H could catalyze the 3′-hydroxylation of different types of flavonoids, and preferred to accept flavanone naringenin, yielding its 3′-hydroxylation product (hesperetin) (Fig. 6). This substrate specificity was consistent with the decrease of 3′-hydroxylated flavonoids and their derivatives after silencing of *CitF3’H* in citrus seedlings. The ER-localization of CitF3’H also underlay its catalytic function in plant. Hence, CitF3’H was a canonical flavonoid 3′-hydroxylase in citrus as most members of the CYP75B subfamily in other plants.

CitF3’H has 99.22% amino acid identity to its orthologous gene (Cs5g11730.1) from *C. sinensis*, with the latter having a 22-amino acid deletion in the N-terminal compared with the former (Fig. S13, see online supplementary material). According to the previous study, *Cs5g11730.1* was a drought-induced gene in citrus and could induce drought tolerance in transgenic *A. thaliana* by enhancing the accumulation of antioxidant flavonoids; however, its catalytic function has not been characterized [40]. Taken this evidence together, CitF3’H and Cs5g11730.1 most probably have the same catalytic function, acting as F3’Hs in citrus, and are likely to be involved in the biosynthesis of 3′-hydroxylated flavonoids both during development and in response to stresses such as UV-light and drought.

It is well known that plant F3’Hs usually belong to the CYP75B subfamily [33]. *CitF3’H* was the sole gene of the CYP75B family in citrus (Table S1, see online supplementary material), thus making it the important gene responsible for flavonoid hydroxylation in citrus fruit peel. However, one gene (CYP98A9) from *A. thaliana* was found to acquire an additional F3’H activity compared with other members of the CYP98A subfamily [35]. Therefore, the possibility of the CYP98A subfamily involved in F3’H activity still cannot be ignored in citrus.

In summary, our work provides a thoroughly genome-wide analysis of the CYP gene superfamily in *C. clementina* genome, identifying 301 CYP genes encoding 319 proteins, which were classified into A-type and non-A-type, including 10 clans grouped into 49 families. We revealed similar exon-intron organizations and motif compositions within the same clan and family, as well as the great divergence between the A-type and non-A-type CYPs, which strongly support the reliability of the phylogenetic relationship. Meanwhile, we demonstrated that frequent duplication events occurred in this CYP superfamily and tandem duplication might have been the major driving force for the rapid expansion. Moreover, our results indicated a wide range of *cis*-acting elements in promoters of CYP genes and elucidated their expression patterns both during development and in response to UV-B. Furthermore, we identified a UV-B-induced CYP gene (*Ciclev10019637m*, designated *CitF3’H*) as a flavonoid 3′-hydroxylase for the first time. We concluded that CitF3’H could catalyze the 3′-hydroxylation of a wide range of flavonoids and preferred to naringenin in yeast cells, and CitF3’H was localized in ER as most CYPs, these results together with the declined content of 3′-hydroxylated flavonoids and their derivatives in the citrus seedlings after silencing of *CitF3’H* support the suggestion that CitF3’H is responsible for the biosynthesis of 3′-hydroxylated flavonoids in citrus. These findings are useful for comprehensively understanding the CYP superfamily in citrus and will facilitate the functional characterization of CYP genes in planta.

## Materials and methods

### Compounds sources

Naringenin, eriodictyol, apigenin, luteolin, liquiritigenin, butin, sakuranetin, kaempferol, quercetin, dihydrokaempferol, dihydroquercetin, eriocitrin, pinocembrin, narirutin, naringin, chrysin, genkwanin, baicalein, scutellarein, norwogonin, wogonin, and neoeriocitrin were purchased from Shanghai Yuanye Bio-Technology Co., Ltd (Shanghai, China). Hesperidin, neohesperidin, sinensetin, isosinensetin, and nobiletin were obtained from Sigma-Aldrich (St Louis, MO, USA). 7-*O*-methyleriodictyol, isosakuranetin, and 5-HPMF were purchased from BioBioPha Co., Ltd (Kunming, China).

### Identification of CYPs in *Citrus clementina*

The genome version of *Citrus clementina* (v1.0) was downloaded from Phytozome v13 (https://phytozome-next.jgi.doe.gov/info/Cclementina_v1_0). To identify putative CYP genes in *C. clementina*, the Hidden Markov Model (HMM) (p450.hmm) corresponding to the conserved domain (PF00067) of CYPs was downloaded from PFAM 35.0 (http://pfam.xfam.org/) and used as queries to perform hmmsearch against the *C. clementina* protein sequences using HMMER 3.2.1 (*e*-value = 0.1) (http://hmmer.org/). In parallel, a local BLASTP search against the *C. clementina* protein database was conducted using amino sequences of CYPs from *A. thaliana* collected from the Cytochrome P450 Homepage [5] as queries (*e*-value = 1e-5). The obtained sequences with protein lengths ranging from 300 to 650 amino acids were further verified via NCBI Conserved Domain Database tool [41]. Finally, a total of 319 transcripts (301 genes) were identified as CYP members in *C. clementina*.

Various physical and chemical parameters of CYPs, including the number of amino acids, molecular weight, theoretical isoelectric points, instability index, aliphatic index, and grand average of hydropathicity, were calculated by ProtParam tool embedded in ExPASy (https://web.expasy.org/protparam/). Additionally, BUSCA web-server was used for predicting the subcellular localization of CYPs (http://busca.biocomp.unibo.it/).

### Phylogenetic analysis, conserved motifs, and gene structures

The protein sequences of citrus CYPs were aligned using MUSCLE [42], and the poorly aligned regions were automatically removed using trimAl [43]. Based on the trimmed alignments, a maximum likelihood (ML) tree was constructed with IQ-TREE [44] and was evaluated with the UltraFast Bootstrap method (5000 bootstrap replicates). Subsequently, the phylogenetic tree was visualized and annotated with iTOL (https://itol.embl.de/).

Conserved motifs of CYPs were identified using MEME (https://meme-suite.org/meme/tools/meme) with the following parameters: number of motifs to find = 15; min motif width = 6; and max motif width = 50. Gene structures of CYPs, including their exon, intron, CDS, and UTR were obtained from the *C. clementina* genome annotation file (GFF3 format). Finally, the conserved motifs and gene structures were visualized using TBtools [45], respectively.

### Collinearity analysis and gene duplication

Collinearity relationship and gene duplication events between citrus CYPs were analysed using the Multiple Collinearity Scan toolkit (MCScanX) with default parameters [46]. The syntenic block and duplicated CYP gene pairs (tandem and segmental duplications) were visualized with shinyCircos [47]. The gene density profile generated by TBtools was also viewed by shinyCircos to display the genome-wide gene density distribution. Non-synonymous (Ka) and synonymous (Ks) substitution rates of duplicated CYP gene pairs were calculated using Simple Ka/Ks Calculator function implemented in TBtools, and the Ka/Ks ratio was used to estimate the selective strength. Additionally, the gene locus of citrus CYPs was mapped to the scaffolds of *C. clementina* genome using TBtools [45].

### *Cis*-acting element analysis in promoters

The 2000-bp region upstream of the initiation codon (ATG) of each citrus CYP transcript was regarded as the promoter sequence. The promoter sequences were extracted using TBtools and subjected to PlantCARE (https://bioinformatics.psb.ugent.be/webtools/plantcare/html/) for the prediction of *cis*-acting regulatory sites. The *cis*-acting elements involved in development, stress, hormone, and light responsiveness were visualized and summarized using TBtools and GraphPad Prism version 7 (GraphPad Software, San Diego, CA, USA).

### Gene expression analysis using RNA-Seq

In our previous study, the fruit peel of ‘Ougan’ cultivar (*Citrus reticulata* cv. *Suavissima*) was used as material, and transcriptome changes in the flavedo during developmental stages (S1, S3, S5, and S7) and in response to UV-B treatment were analysed using RNA-Seq [28]. In this study, expression data of citrus CYP genes were retrieved from these transcriptomic data and processed as FPKM (fragments per kilobase of exon per million fragments mapped) values. Genes were clustered based on their expression levels using Mfuzz (cluster number = 9) [48]. Expression heatmaps were drawn using TBtools [45].

### Protein expression and enzyme assays in a yeast system

The full-length of *CitF3’H* (*Ciclev10019637m*) and *Ciclev10033591m* without the termination codon were cloned into pYES2/NT C vector using primers listed in Table S6 (see online supplementary material). Recombinant constructs or an empty vector were transformed into yeast strain INVSc1 (*Saccharomyces cerevisiae*) via the Quick and Easy Yeast Transformation Mix kit (Takara, Dalian, China). The transgenic yeast cells were initially cultured in a 10 mL synthetically defined medium lacking uracil (SD-Ura) liquid medium (Takara, Dalian, China) supplemented with 2% glucose at 30°C for 24 h with shaking at 200 rpm. Yeast cells were then harvested by centrifugation 1500 × g for 5 min before being resuspended in an equal volume (10 mL) of induction medium (SD-Ura liquid medium containing 2% galactose). Flavonoid substrates were added to the cultures (incubated to OD_600_ = 1.2) at a final concentration of 20 μM. After 24 h of incubation, the cultures were extracted twice with an equal volume of ethyl acetate, and 8 mL of the upper organic phase was dried and resolved in 200 μL methanol for HPLC or HPLC-MS/MS.

The relative activity of CitF3’H was measured by scaling up the procedure described above. Specifically, large-scale induced yeast cultures (200 mL) were prepared for the determination of enzyme activity toward various flavonoid substrates. For each substrate, 5 mL of the induced yeast cultures were set as a replicate, and a total of three replicates were used in enzyme assays. For substrate bias evaluation, the enzyme activity was evaluated by controlling the conversion of substrate at less than 10% of the total substrate. In detail, after 10 h of incubation with the corresponding substrate, the cultures were extracted twice with an equal volume of ethyl acetate, and 6 mL of the organic phase was dried and resolved in 200 μL methanol for HPLC analysis. The measured velocity was close to the true initial velocity, which could be used to calculate the enzyme activity.

### Virus-induced gene silencing in citrus

Gene-silenced plants were generated via tobacco rattle virus (TRV)-based VIGS as described previously [49]. The germinating seeds of Ponkan (a citrus cultivar with abundant 3′-hydroxylated flavonoids) were subjected to infiltration in the experiment. A 326-bp fragment of CDS from *CitF3’H* was cloned into the TRV2 vector to invoke efficient gene silencing by agroinoculation. *Agrobacterium* (EHA105) cultures (OD_600_ = 1.2) carrying TRV1 and TRV2 were centrifuged and resuspended in an equal volume of infiltration buffer (10 mM 2-(*N*-morpholino) ethanesulfonic acid, 10 mM MgCl_2_, 150 μM acetosyringone, pH = 5.6) and mixed at a 1:1 (vol/vol) ratio. These *Agrobacterium* suspensions were infiltrated into germinating seeds with sprouts ~1 cm in length via a SHB-IIIA vacuum chamber (−100 kPa, 1 min) (Shanghai Yukang Science and Education Equipment Co., Ltd, Shanghai, China). Infiltrated sprouts were rinsed with water and sown in Murashige & Tucker (MT) solid medium (PhytoTechnology Laboratories, Shawnee Mission, KS, USA) in darkness for three days, followed by growing in soil in a growth chamber (Zhejiang Qiushi Artificial Environment Co., Ltd, Hangzhou, China) for one month. Plants co-inoculated with TRV1 and TRV2 were used as vector control. Aerial parts of each plant were sampled for further analysis. Primers used in the construction of *CitF3’H*-TRV2 are listed in Table S6 (see online supplementary material).

### RNA isolation and qRT–PCR

For the samples in the VIGS experiment, total RNA isolation and cDNA synthesis were performed as described previously [28]. Real-time quantitative reverse transcription–PCR (qRT–PCR) of *CitF3’H* was carried out on a Bio-Rad CXF96 instrument (Bio-Rad, Hercules, CA, USA) with a TB Green *Premix Ex Taq* (Tli RNaseH Plus) kit (Takara, Dalian, China) according to the manufacturer’s instructions. The relative expression of *CitF3’H* was calculated using the 2^-ΔCt^ method, using the citrus β-actin gene as the housekeeping gene [50]. Primers used for qRT–PCR are shown in Table S6 (see online supplementary material).

### Metabolite analysis

HPLC and MS/MS analyses were performed to analyse metabolites in enzyme assays and plant materials. For the latter, samples were prepared as described previously [28].

HPLC analysis was performed on a Waters 2695 HPLC system (Waters Corp, Milford, MA, USA) equipped with a Sunfire C18 ODS column (4.6 × 250 mm, 5 μm), quaternary solvent manager and a 2998 PDA detector. Separation was conducted using water (A) and acetonitrile (B) with the following gradient: 0–5 min, 20% B; 5–10 min, 20%–27% B; 10–15 min, 27% B; 15–25 min, 27%–40% B; 25–35 min, 40%–60% B; 35–40 min, 60%–80% B; 40–42 min, 80%–100% B; 42–45 min, 100%–20% B; 45–50 min, 20% B. The flow rate was set as 1 mL·min-1 and the injection volume was 10 μL. The temperature of the column and samples were maintained at 25°C and 8°C, respectively. Metabolites were detected at the wavelength of 200–400 nm.

MS/MS was conducted on an AB TripleTOF 5600plus System (AB SCIEX, Framingham, MA, USA). MS2 spectra were obtained in positive ion mode (ESI) or negative ion mode and the exact mass was measured.

### Subcellular localization assays

The CDS of *CitF3’H* without stop codon was cloned into the *35S*-eGFP vector, then transferred to *Agrobacterium* strain (GV3101: pSoup). *Agrobacterium* cells harboring *CitF3’H*-GFP and mCherry-labeled ER-marker (ER-rk *CD3–959*) [51] were co-infiltrated at a 1:1 (vol/vol) ratio in leaves of 4 weeks old tobacco (*Nicotiana tabacum*) as described previously [52]. The corresponding GFP (empty vector) was used as a positive control. After three days, tobacco leaves containing the corresponding vector were imaged to observe the green fluorescent protein (GFP) and mCherry fluorescence with a Zeiss LSM710NLO confocal laser scanning microscope. Primers for the construction of *CitF3’H*-GFP are described in Table S6 (see online supplementary material).

## Supplementary Material

Web_Material_uhac283

## Data Availability

The data used in this study are included in the article.
