# Supplementary material for: Genome-wide analysis of cytochrome P450 genes in Citrus clementina and characterization of a CYP gene encoding flavonoid 3′-hydroxylase
Source: Hortic Res. 2022 Dec 23;10(2):uhac283. doi: 10.1093/hr/uhac283 (PMC9930397; doi:10.1093/hr/uhac283)
Supplement: Web_Material_uhac283 [file web_material_uhac283.zip › supplementary Figures S1-S13.pdf]

Supplementary data

Supplementary data is available at Horticulture Research Journal online.

Figure S1 Schematic diagram of conserved domains in citrus CYPs. The protein sequence of each CYP was verified by the NCBI Conserved Domain Database tool [41].

Figure S2 Schematic diagram of conserved motifs in citrus CYPs. Different motifs are represented by different colors.

Figure S3 WebLogos of the 13 conserved motifs in citrus CYPs. Each letter represents an amino acid and its height corresponds to the relative frequency.

Figure S4 Schematic diagram of gene structures in citrus CYPs. Blue and yellow rectangles represent untranslated regions (UTR) and coding sequences (CDS), respectively. The lines without any rectangles represent introns.

Figure S5 Distribution of citrus CYPs in 11 scaffolds. The number of CYP genes in each scaffold is indicated above the scaffold. Tandem duplications are indicated by arcs.

Figure S6 Schematic diagram of four categories of *cis*-acting elements in the promoter of citrus CYP genes.

Figure S7 Heatmap display of the number of each *cis*-acting element in the promoter of citrus CYPs.

Figure S8 Expression of CYP genes in the flavedo of citrus during development. (A) Genes were grouped into nine distinct clusters based on their expression patterns (B) and mapped to ten CYP clans. Expression values were obtained from our previous study [28] and can be found in Supplementary Table 1.

Figure S9 Expression of CYP genes in the flavedo of citrus in response to UV-B irradiation. Expression levels of four responsive patterns of the CYP genes in response to UV-B irradiation for 24 h and 48 h are represented as four heatmaps, including the up-regulated genes (A), down-regulated genes (B), down-regulated after irradiation for 24 h and up-regulated after irradiation for 48 h (C), and up-regulated after irradiation for 24 h and down-regulated after irradiation for 48 h (D). Among the up-regulated genes, 17 CYP genes belonging to flavonoid-related families are indicated in red font. The mean expression value of each gene was automatically scaled and visualized as heatmaps by TBtools. Expression values were obtained from our previous study [28] and can be found in Supplementary Table 1.

Figure S10 HPLC chromatograms of yeast cultures expressing Ciclev10033591m with naringenin (A) and apigenin (B) as substrates. Top, authentic compounds of substrates and their 3'-hydroxylated

derivatives; middle, yeast harboring the empty vector; bottom, yeast expressing Ciclev10033591m. Nar, naringenin; Eri, eriodictyol; Api, apigenin; Lut, luteolin.

Figure S11 MS/MS data of the new peaks produced by CitF3'H in yeast cells and their authentic compounds. Substartes used were naringenin (A), sakuranetin (B), liquiritigenin (C), apigenin (D), kaempferol (E) and dihydrokaempferol (F). Analyses were operated in positive ion mode (A, D and E) or negative ion mode (B, C and F).

Figure S12 Chemical structural formulas of the substrates that could not be catalyzed by CitF3'H in yeast cells.

Figure S13 Sequence alignment of CitF3'H (accession number: Ciclev100019637m) and its orthologous gene (accession number: Cs5g11730.1) in *Citrus sinensis* at the protein level.

Table S1 Comprehensive information of the CYP superfamily in citrus. Detailed information is as follows: the accession number of genes, mRNAs and proteins; the located scaffold, gene range and gene length; the classification name designated by the previous study [23] and the CYP nomenclature system; the assigned family and clan by the CYP nomenclature system and phylogenetic tree; the number of exon, intron, CDS and UTR, conserved motif and *cis*-acting element in each gene; the expression levels of CYP genes in the flavedo of citrus both during development (including the cluster number by Mfuzz) and in response to UV-B irradiation; various physical and chemical properties, including the number of amino acid, molecular weights, theoretical isoelectric points, subcellular localizations, instability index, aliphatic index and grand average of hydropathicity.

Table S2 Number of citrus CYP genes and transcripts in clans and families.

Table S3 Classification of duplication origin information for all citrus CYP genes. A sum of four duplication types was identified using MCScanX, including WGD (whole genome) or segmental (i.e. collinear genes in collinear blocks); tandem (consecutive repeat), Proximal (in nearby chromosomal region but not adjacent) and dispersed (other modes than segmental, tandem and proximal) duplications.

Table S4 Tandem and segmentally duplicated gene pairs in citrus CYPs. The red font indicates the genes with both tandem and segmental duplication events. The Ka/Ks ratio of each duplicated pair was also calculated.

Table S5 Function and category of each *cis*-acting element identified in the promoter of citrus CYPs.

Table S6 Primers used for recombinant constructs and qRT–PCR.

Supplementary Figure S1

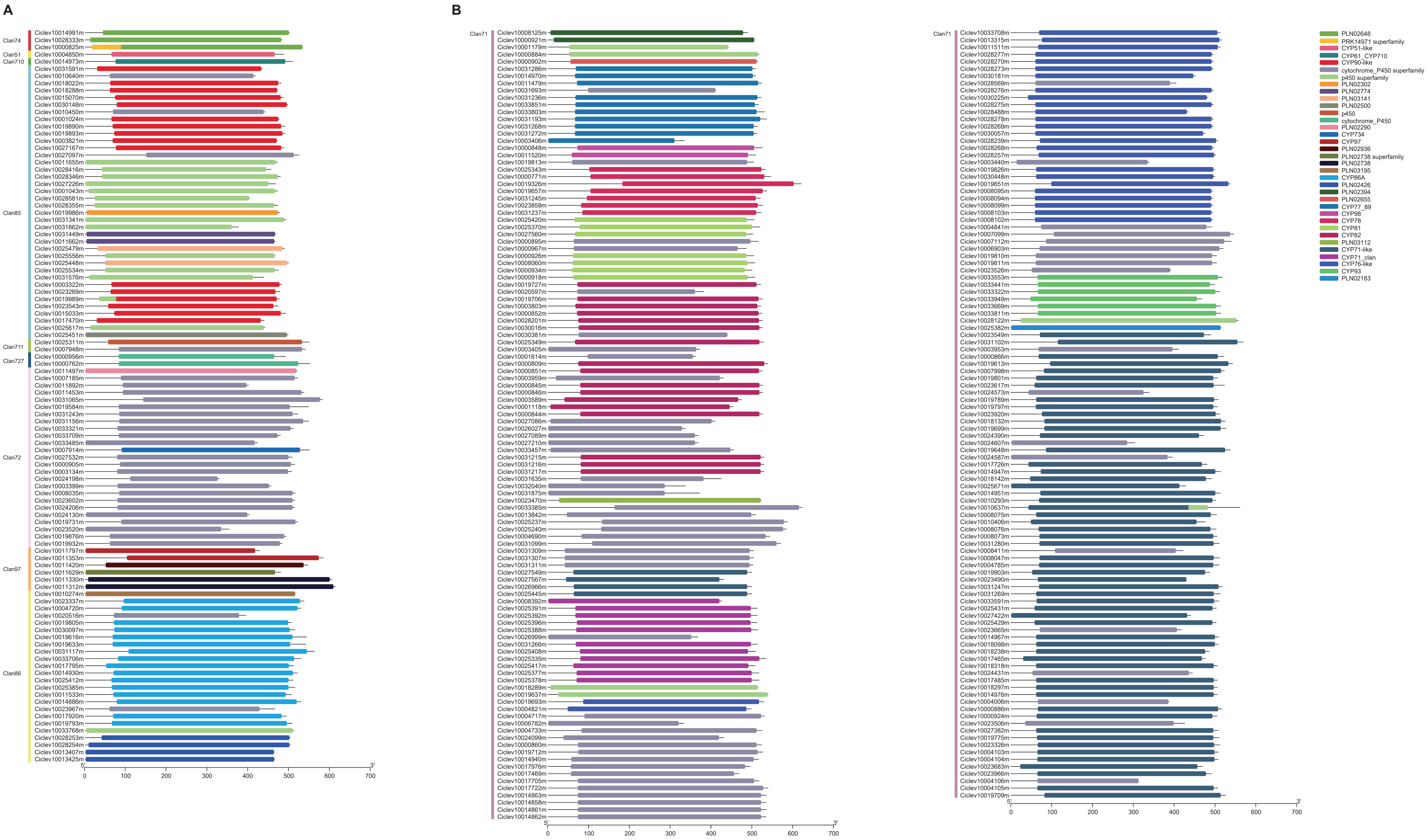

Supplementary Figure S2

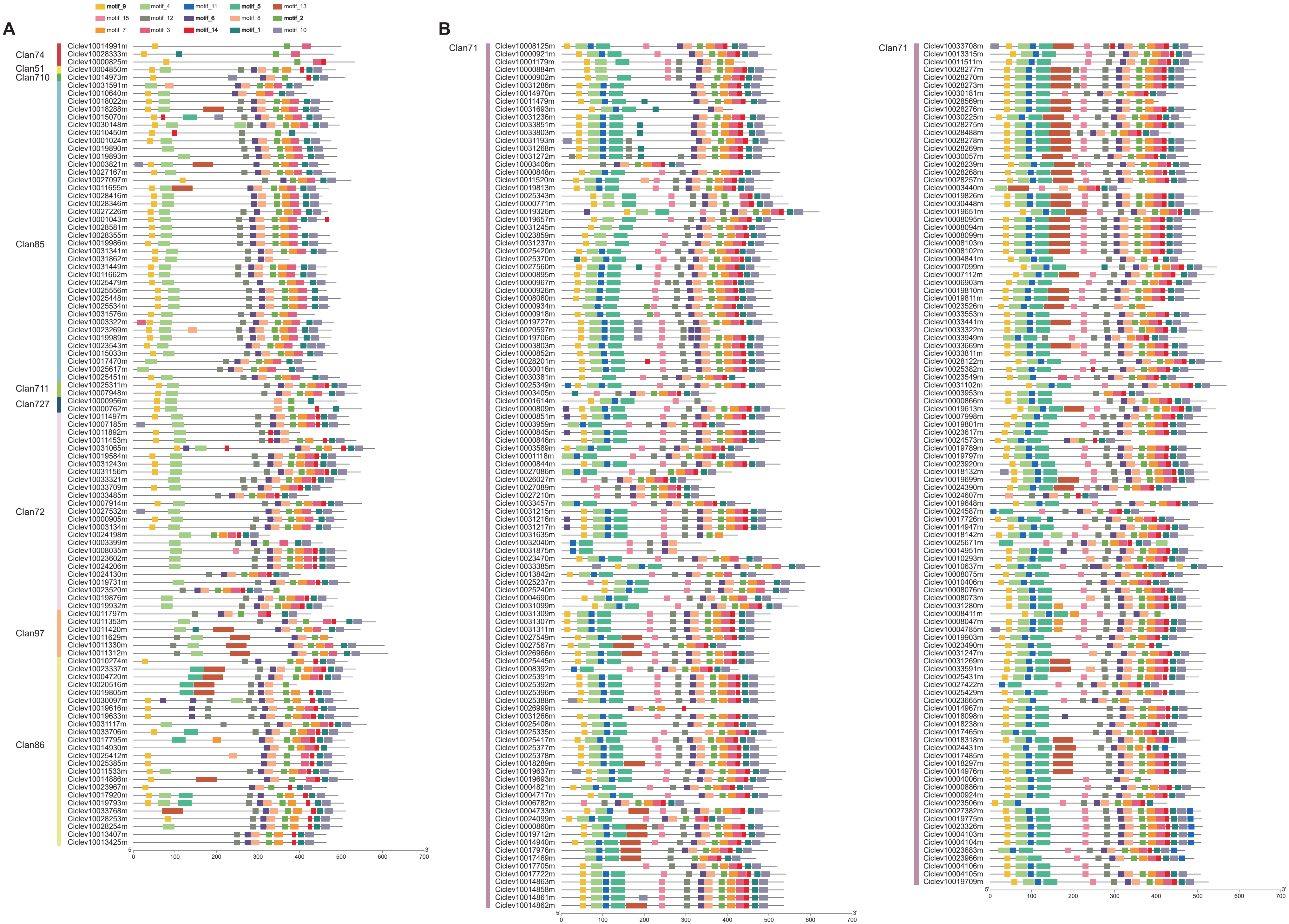

Supplementary Figure S3

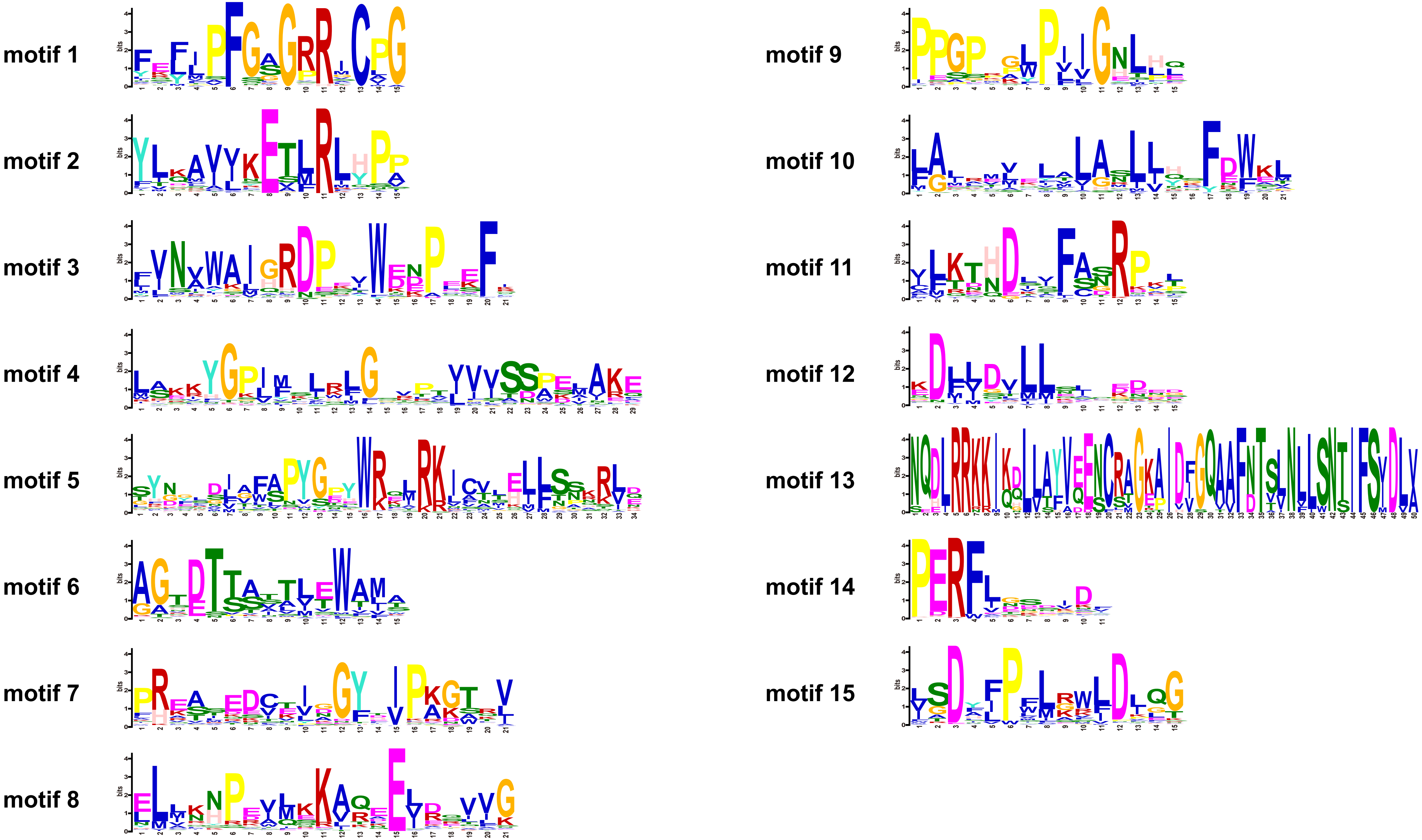

Supplementary Figure S4

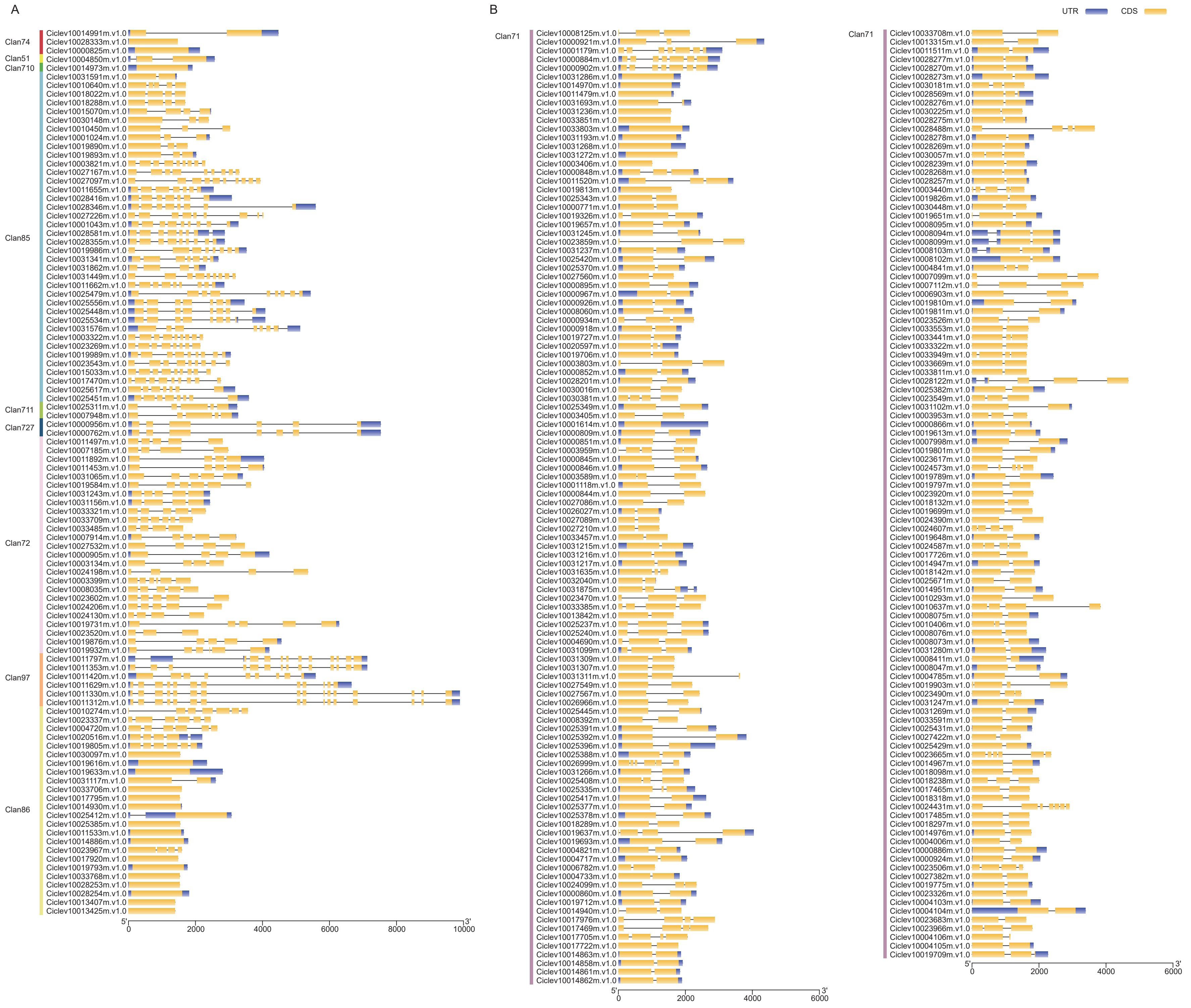

Supplementary Figure S5

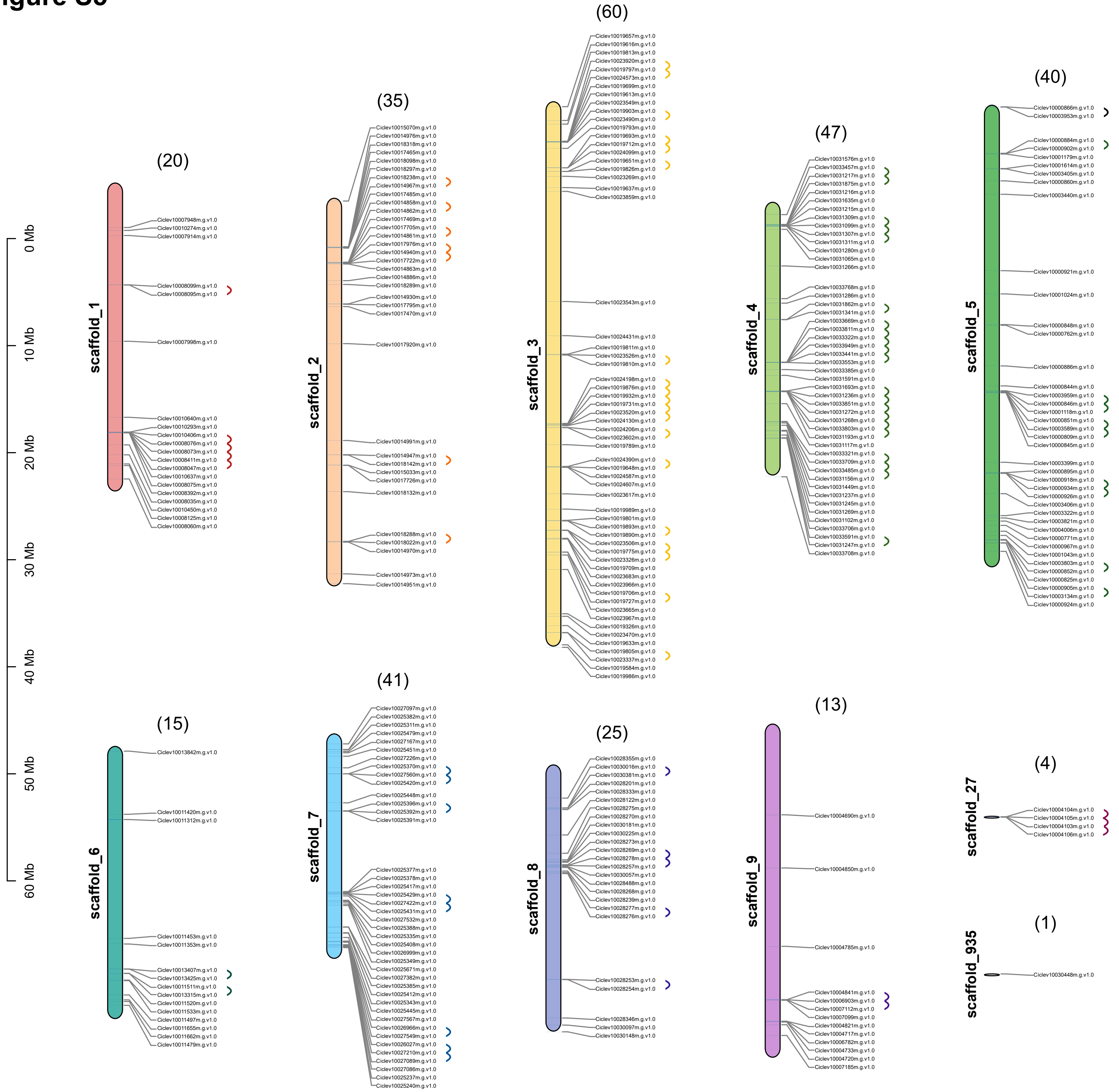

Supplementary Figure S6

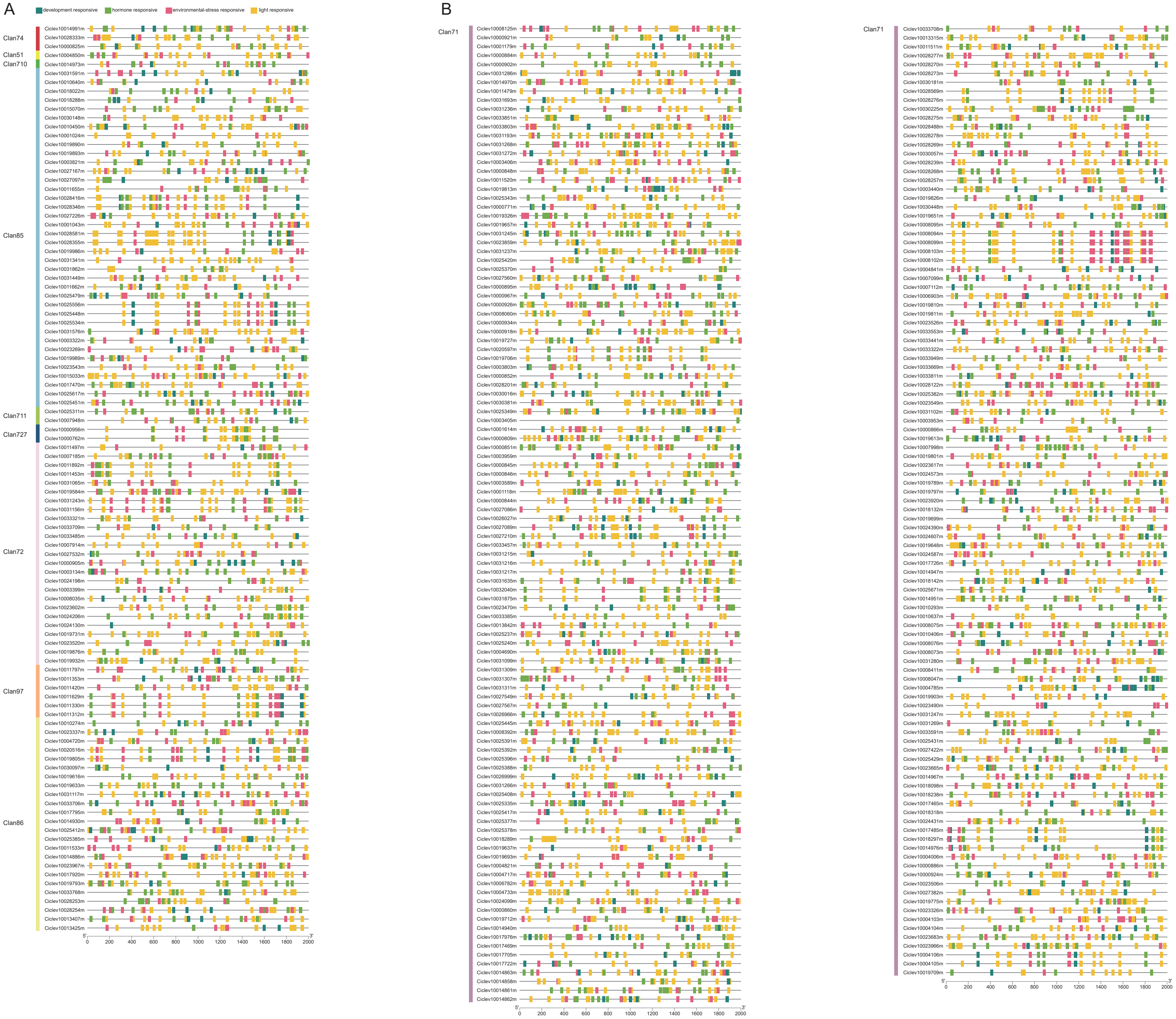

Supplementary Figure S7

A

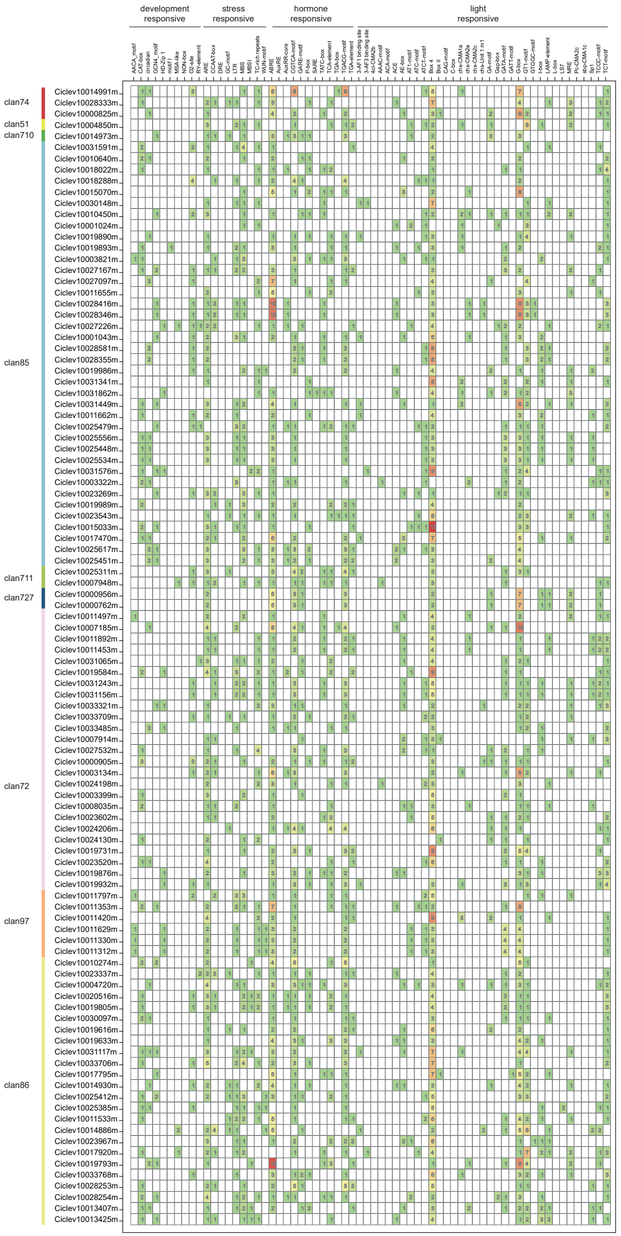

B

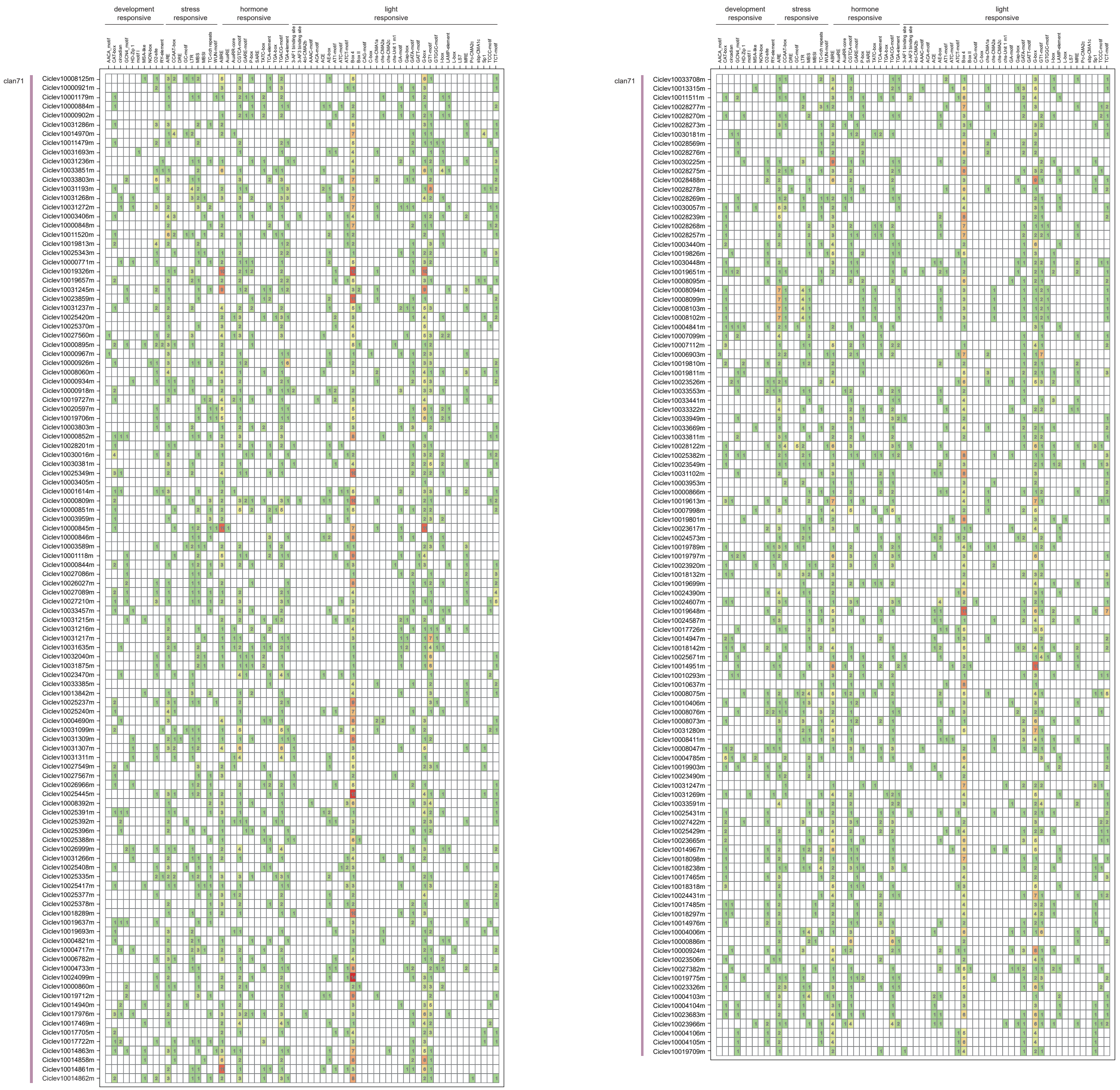

Supplementary Figure S8

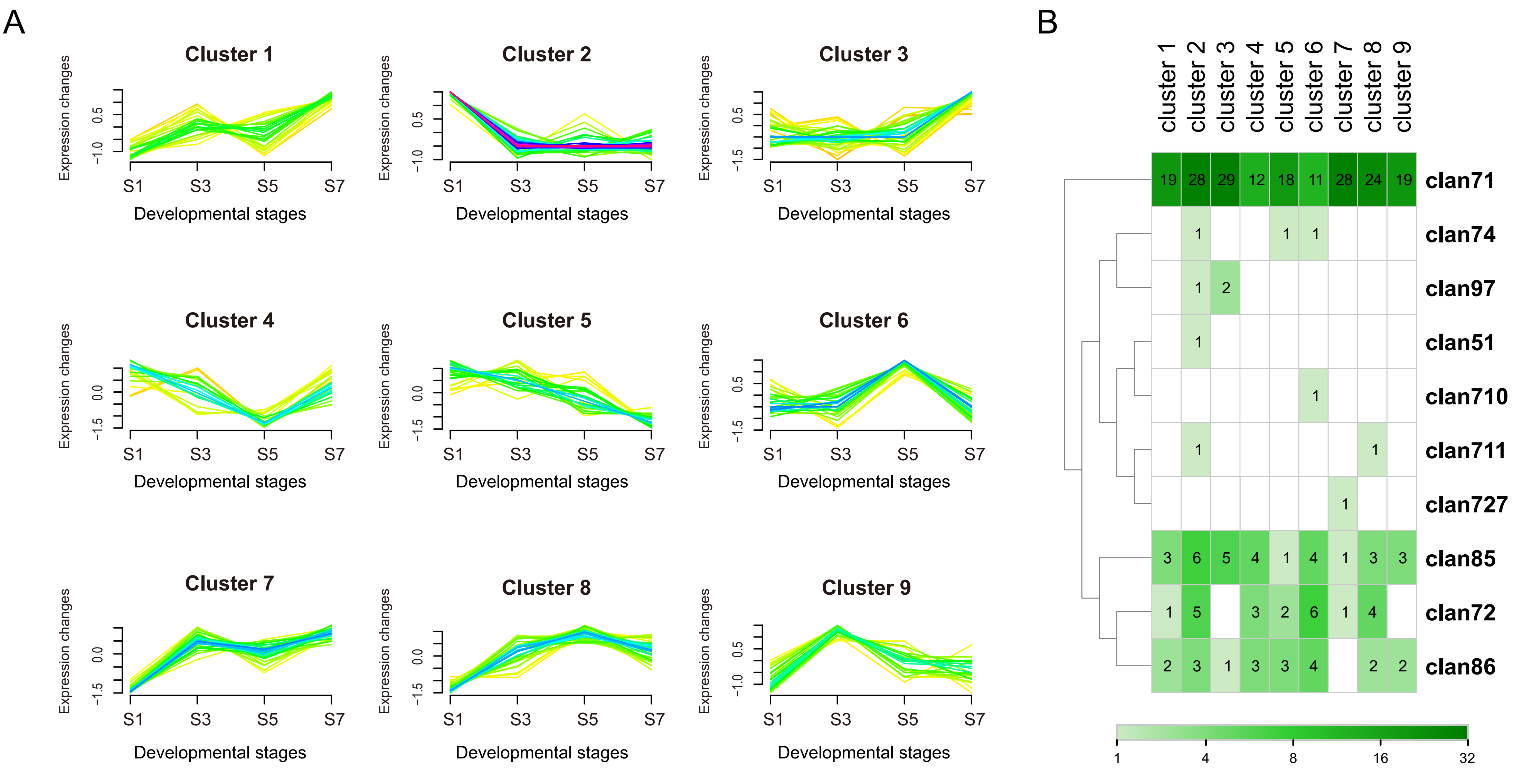

Supplementary Figure S9

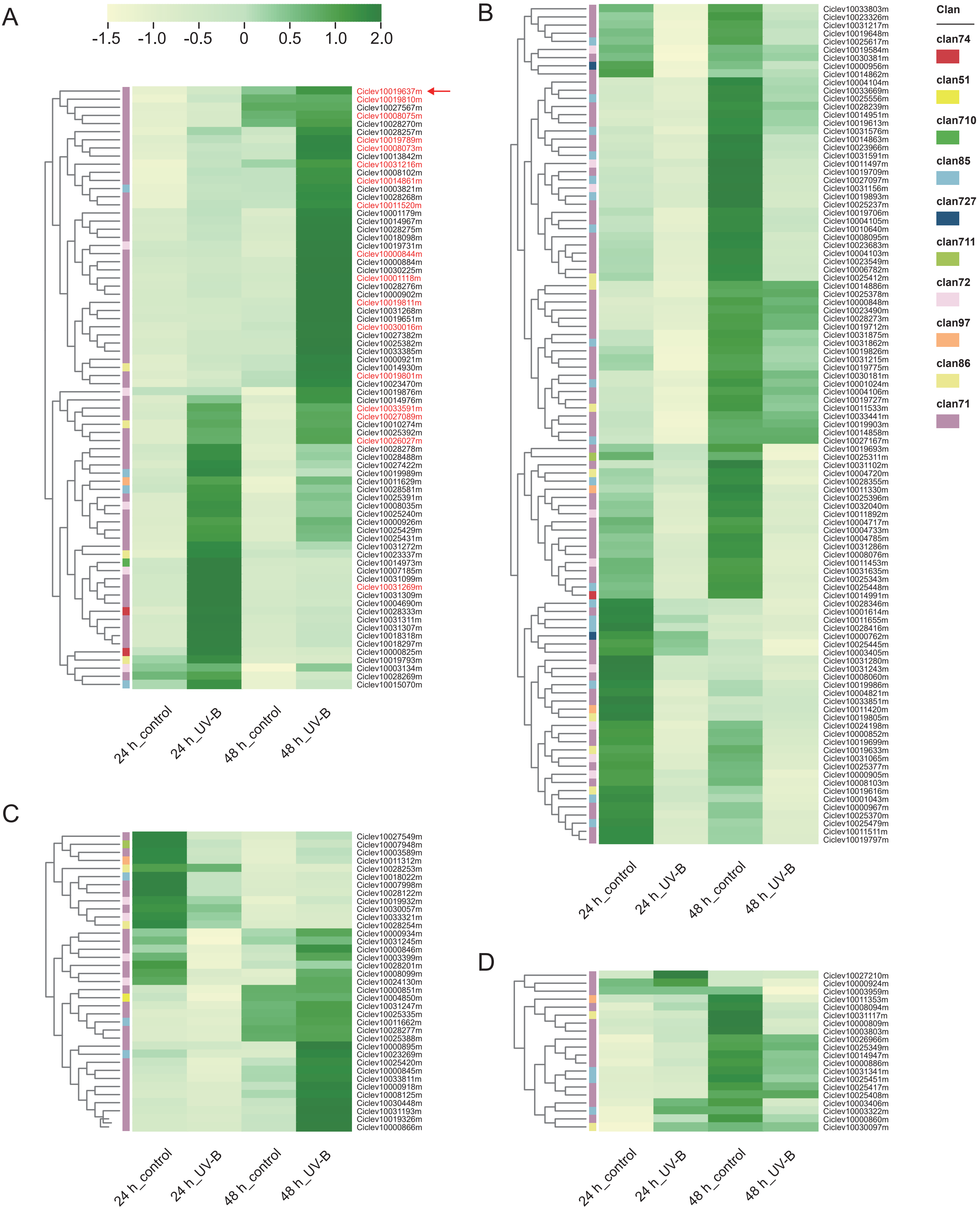

Supplementary Figure S10

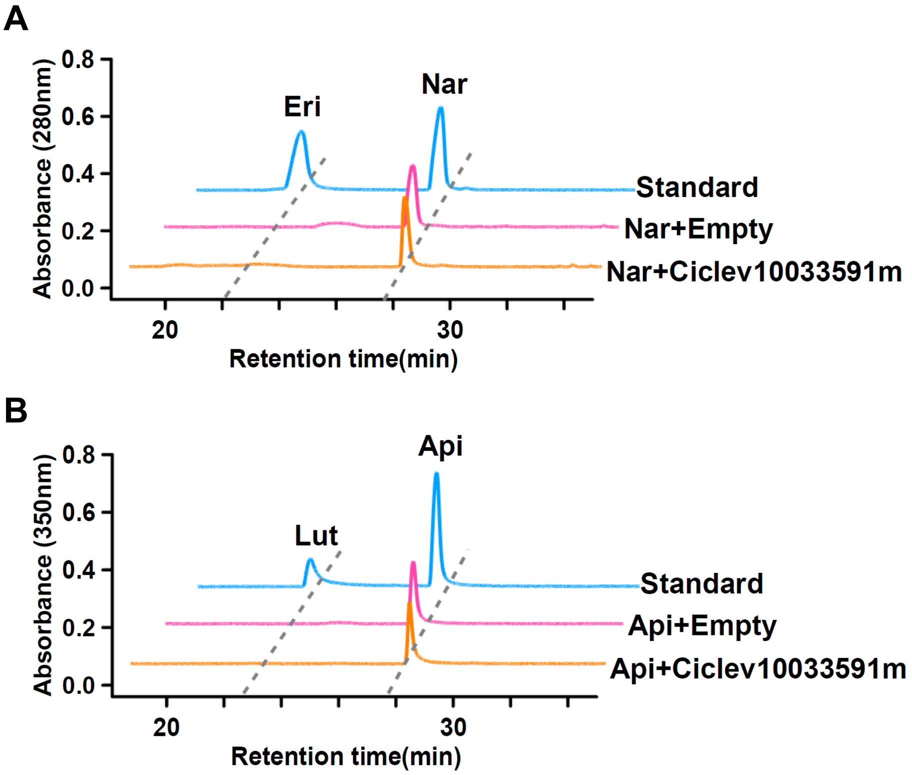

Supplementary Figure S11

A naringenin hydroxylated product by CitF3'H

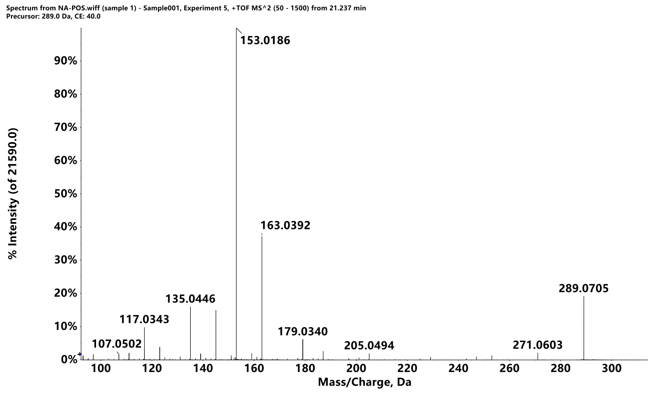

eriodictyol standard

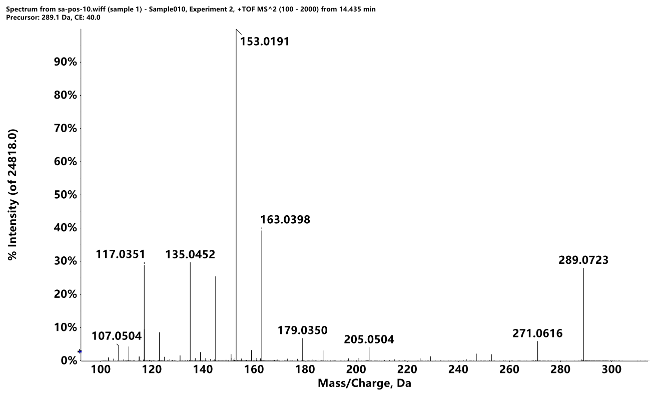

B sakuranetin hydroxylated product by CitF3'H

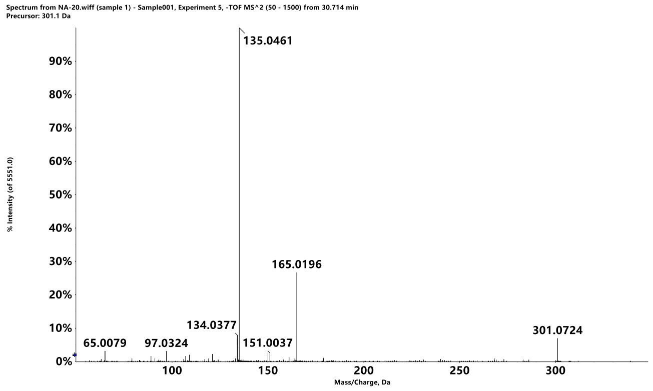

7-*O*-methyleriodictyol standard

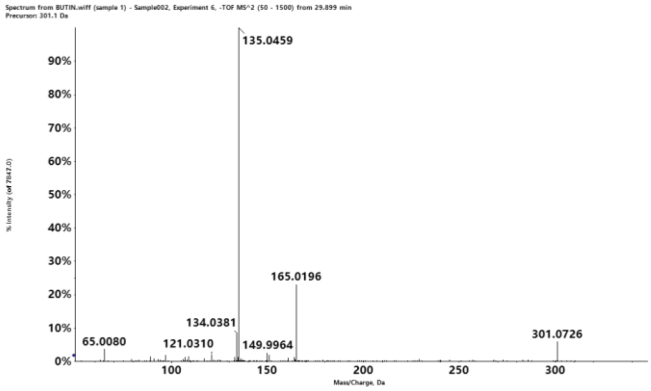

C liquiritigenin hydroxylated product by CitF3'H

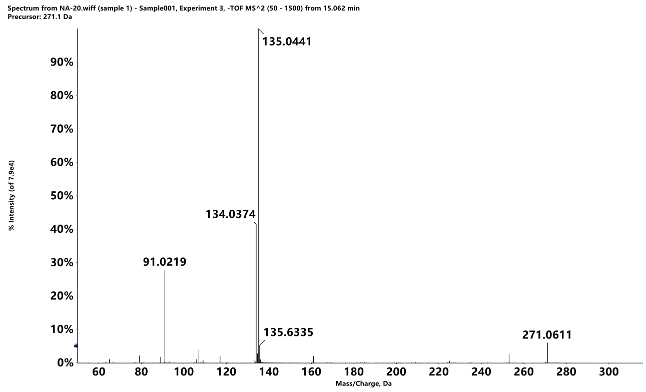

butin standard

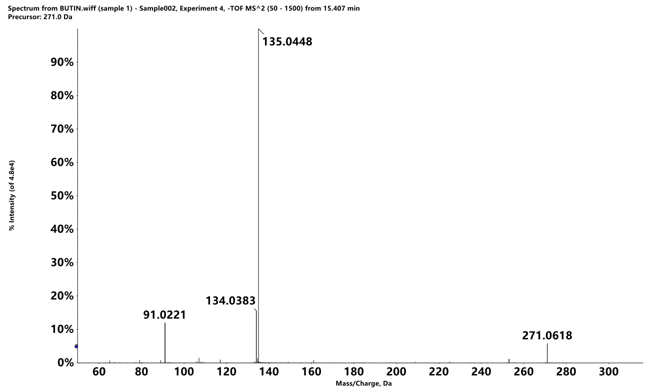

D apigenin hydroxylated product by CitF3'H

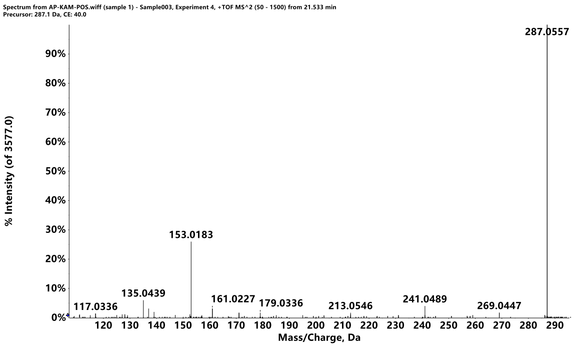

luteolin standard

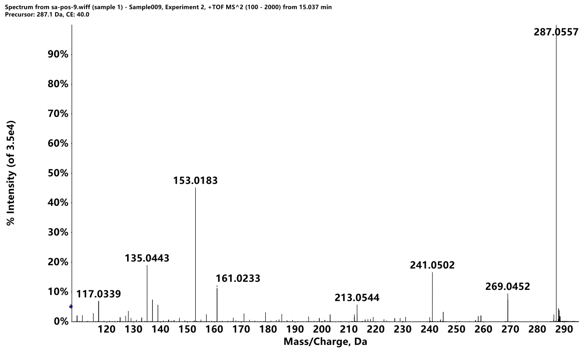

E kaempferol hydroxylated product by CitF3'H

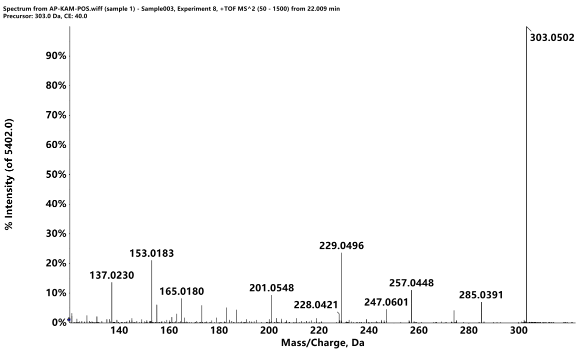

quercetin standard

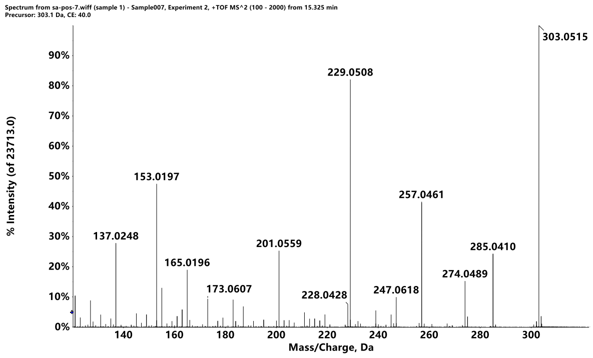

F dihydrokaempferol hydroxylated product by CitF3'H

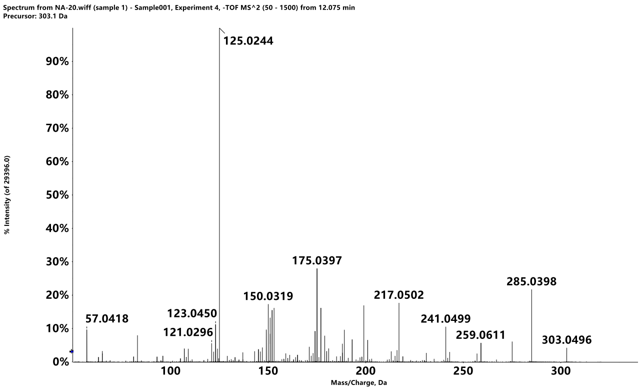

dihydroquercetin standard

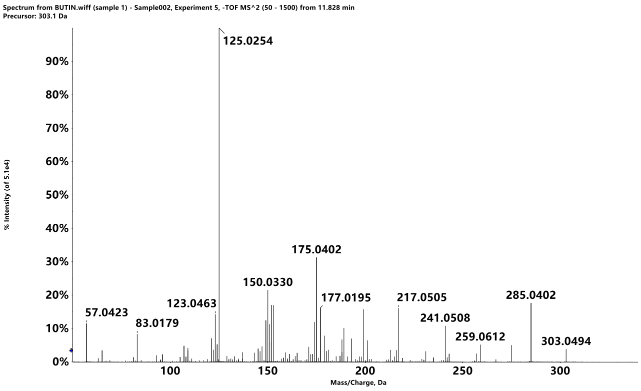

Supplementary Figure S12

Flavanone

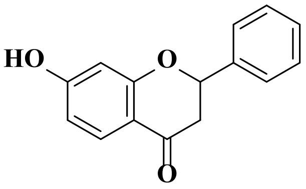

pinocembrin

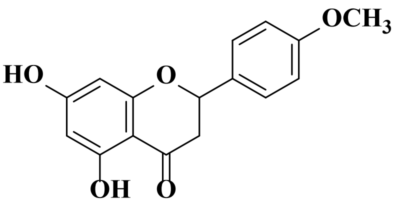

isosakuranetin

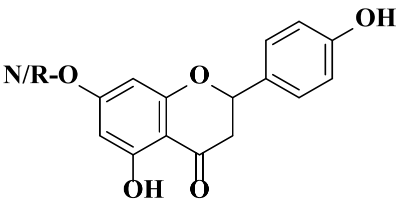

naringin / narirutin

Flavone

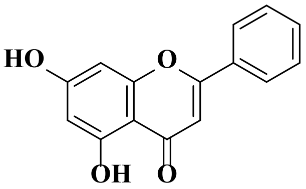

chrysin

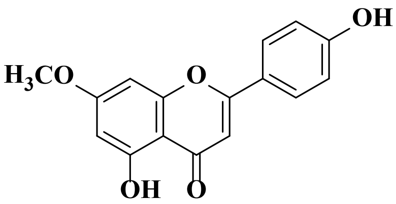

genkwanin

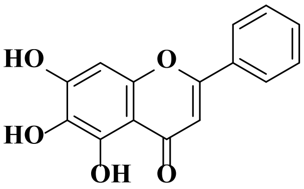

baicalein

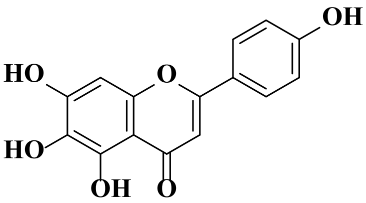

scutellarein

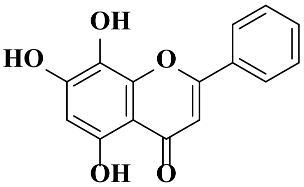

norwogonin

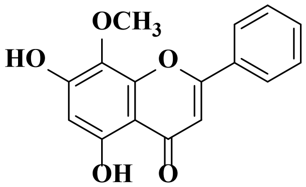

wogonin

Supplementary Figure S13

```
Cs5g11730.1         ----------------------MSTLPLLILYTSLLAIVISFLFSLLRNRRRHSSHRLPP    38
Ciclev10019637m     MQPLVRLLVPFLRFLWETKQRAMSTLPLLILYTSLLAIVISFLFSLLRNRRRHSSHRLPP    60
                                          **************************************

Cs5g11730.1         GPKPWPIVGNLPHLGPMPHQSIAGLARTHGPLMYLRLGFVDVVVAASASVAAQFLKIHDS    98
Ciclev10019637m     GPKPWPIVGNLPHLGPMPHQSIAGLARTHGPLMYLRLGFVDVVVAASASVAAQFLKIHDS    120
                    ************************************************************

Cs5g11730.1         NFSNRPPNSGAKHIAYNYQDIVFRPYGPRWRMLRKISSVHLFSGKALDDYRHVRQEEMAV    158
Ciclev10019637m     NFSNRPPNSGAKHIAYNYQDIVFRPYGPRWRMLRKISSVHLFSGKALDDYRHVRQEEMAV    180
                    ************************************************************

Cs5g11730.1         LARALASAGTEPVNLAQRLNLCVVNALGRVMLGFRVFGDGTGGSDPRADEFKSMVVELMV    218
Ciclev10019637m     LTRALASAGTEPVNLAQRLNLCVVNALGRVMLGFRVFGDGTGGSDPRADEFKSMVVELMV    240
                    *:**********************************************************

Cs5g11730.1         LAGVFNVGDFVPALERLDLQGVARKMKKLHKRFDVFLSDILEERKMNGRDGGNKHTDLLG    278
Ciclev10019637m     LAGVFNVGDFVPALERLDLQGVARKMKKLHKRFDVFLSDILEERKMNGRDGGNKLTDLLG    300
                    ****************************************************** *****

Cs5g11730.1         TLISLMDDANGEEKLTETEIKALLLNMFTAGTDTSSSTIEWAIAELIRHPKVRAQVQQEL    338
Ciclev10019637m     TLISLMDDANGEEKLTETEIKALLLNMFTAGTDTSSSTIEWAIAELIRHPKVWAQVQQEL    360
                    **************************************************** *******

Cs5g11730.1         DSVVGRDRLVTELDLPQLTYLQAVIKEIFRLHPSTPLSLPRAASESCKINGYDIPKGSTL    398
Ciclev10019637m     DSVVGRDRLVTELDLPQLTYLQAVIKEIFRLHPSTPLSLPRAASESCKINGYDIPKGSTL    420
                    ************************************************************

Cs5g11730.1         LVNIWAIARDPNEWADPLEFRPERFLPGGEKYNVDVKGNDYELIPFGAGRRICAGLSWGL    458
Ciclev10019637m     LVNIWAIARDPNEWADPLEFRPERFLPGGEKYNVDVKGNDYELIPFGAGRRICAGLSWGL    480
                    ************************************************************

Cs5g11730.1         RMVQLGTATLAHAFNWELPGGLKPEKLSMDEAYGLTLQRAAPLVVHPRPRLSPNAYQA*     516
Ciclev10019637m     RMVQLGTATLAHAFNWELPGGLKPEKLNMDEAYGLTLQRAAPLVVHPRPRLSPNAYQA*     538
                    ***************************.******************************
```
